# Isolation, structure revision and stereochemistry of trichomycins A and B, aromatic analogues of amphotericin B

**DOI:** 10.1038/s41598-025-28626-x

**Published:** 2025-12-29

**Authors:** Tomasz Laskowski, Filip Anaszewicz, Julia Borzyszkowska-Bukowska, Julia Pakuła, Małgorzata Michałowska, Julia Bublewska, Dorota Gudanis-Sobocińska, Katarzyna Kozłowska-Tylingo, Paweł Szczeblewski

**Affiliations:** 1 Department of Pharmaceutical Technology and Biochemistry and BioTechMed Centre, Faculty of Chemistry, Gdansk University of Technology, Gabriela Narutowicza Str. 11/12, Gdańsk, 80–233 Poland; 2 Department of Biomolecular NMR, Institute of Bioorganic Chemistry Polish Academy of Sciences, Zygmunta Noskowskiego Str. 12/14, Poznań, 61–704 Poland

**Keywords:** Biochemistry, Chemical biology, Chemistry, Computational biology and bioinformatics, Drug discovery, Microbiology

## Abstract

Fungal infections pose an increasing global health threat, particularly to immunocompromised individuals. In response, the World Health Organization has prioritized the development of new antifungal agents. Among clinically used polyene macrolides, trichomycin remains structurally ambiguous despite decades since its discovery. This study presents an integrated re-assessment of the trichomycin complex focused on its two principal constituents using a combination of liquid chromatography–mass spectrometry (HPLC–DAD–ESI-MS), preparative-scale HPLC, two-dimensional NMR spectroscopy, and computational modeling. Four principal components were identified, including trichomycins A and B and their respective all-trans isomers, formed via light-induced isomerization. One-step prep-HPLC isolation enabled the purification of both trichomycins in analytically pure form. Detailed NMR analysis redefined the polyol fragment (C1–C14) of trichomycin A and confirmed structural consistency in trichomycin B. Absolute configurations of 13 stereocenters in each molecule were assigned by integrating experimental ³J coupling constants, ROE data, and molecular modeling, incorporating the generalized Karplus equation. The chromophore geometry was determined as 22*E*, 24*E*, 26*E*, 28*Z*, 30*Z*, 32*E*, 34*E*. By resolving key structural inconsistencies, this work lays the foundation for the rational derivatization of trichomycin and supports future efforts toward the development of novel polyene-based antifungal agents with improved pharmacological profiles.

**Supplementary Information:**

The online version contains supplementary material available at 10.1038/s41598-025-28626-x.

## Introduction

Modern medicine faces many challenges. For years, an uneven battle has been carried out against cancer, with many lives lost and many more affected. The recent years have been marked by significant disruption due to the COVID-19 pandemic. Infections caused by fungal pathogens are another threat that accompanies both oncology patients and those struggling with infections that weaken the immune system. In 2022, the World Health Organization (WHO) published a list of priority fungal pathogens considered to pose a substantial threat to public health^1^. One of the key strategies recommended by WHO to mitigate the burden of fungal diseases is the focused support of research and innovation aimed at accelerating the development of new antifungal agents and improving diagnostic methods. Achieving this goal involves both the identification of novel molecular targets in fungal pathogens and the structural modification of existing compounds based on the known frameworks of effective antifungal agents, enabling the development of more selective and potent therapies^2–8^.

The listed pathogens were categorized into three groups based on the level of threat they pose: *critical*, *high*, and *moderate*. Of particular concern are species of the genus *Candida* — including *Candida albicans*, *C. auris*, and *C. glabrata* — which were classified as critical priority pathogens.

Polyene macrolides, produced as secondary metabolites by *Streptomyces* spp., represent the first clinically applied antifungal agents and are defined by macrocyclic lactone scaffolds bearing conjugated polyene systems and hydrophilic substituents, conferring potent activity against *Candida* species^9–11^.

Polyene macrolides exhibit a broad antifungal spectrum and high potency, including activity against multidrug-resistant fungal strains^12–15^. Moreover, resistance to this class of antibiotics remains exceptionally rare^16^. These features render polyenes among the most promising scaffolds for developing novel agents to treat life-threatening systemic fungal infections^17,18^. Six polyene antifungals—nystatin, amphotericin B (AmB), natamycin, methyl partricin, candicidin, and trichomycin—have found clinical use^13^.

For over six decades, scientists have attempted to elucidate the structures of these compounds, a fundamental step toward understanding their molecular mechanisms of action and enabling rational drug design to improve their therapeutic properties^19,20^.

Despite the long-standing clinical use of polyene macrolides, numerous inaccuracies and ambiguities persist in the scientific literature regarding their structural and biochemical properties^21–23^. One notable example is trichomycin, a member of the aromatic heptaene macrolides (AHM).

Originally developed by Fujisawa Pharmaceutical Company, trichomycin — also known as hachimycin (International Nonproprietary Name), listed in the Japanese Pharmacopoeia (JP18) — has been used primarily for treating vaginal infections (candidiasis, trichomoniasis), urinary tract infections caused by *Candida*, and fungal keratitis (administered in eye drop form). In the international Anatomical Therapeutic Chemical (ATC) Classification System, trichomycin is registered under three codes: D01AA03 (topical antifungal for skin and mucosal infections), G01AA06 (intravaginal antimicrobial for candidiasis and trichomoniasis), and J02AA02 (systemic antifungal for gastrointestinal and disseminated fungal infections^24^.

Trichomycin was first described in 1952 and was isolated from *Streptomyces hachijoensis*^25,26^. Initially believed to consist of just two components — trichomycin A and B — it is now recognized as a complex mixture of structurally related compounds. Using HPLC, Helboe et al. (1980) estimated that the complex contains at least ten components, while Thomas and Newland (1986) proposed as many as sixteen^27,28^. However, these findings were not followed up by preparative-scale isolation. More comprehensive studies by Komori and Morimoto (1989) revealed that the mixture consists of at least seventeen individual compounds. They successfully isolated six major components — trichomycins A through F — which constitute approximately 70% of the complex^29^.

The biological activity of trichomycin and its main constituents has been investigated in both in vitro assays and clinical settings. A 1955 study by Magara et al. demonstrated the clinical efficacy of trichomycin in treating recurrent vulvovaginitis caused by various *Candida* species. A four-day regimen of 50 mg/day (administered both orally and intravaginally) led to a nearly complete resolution of symptoms, with no observed side effects or toxicity^30^.

Biological activity assays further revealed that trichomycin A was substantially more effective than amphotericin B against *Candida spp.* and *Trichomonas vaginalis*, although amphotericin B remained superior against *Aspergillus* species^29,31^. In contrast, trichomycin B exhibited markedly lower bioactivity — approximately eightfold less than trichomycin A — but still outperformed miconazole in antifungal and antiprotozoal assays^29,32^.

Among the identified components of the antibiotic complex, trichomycins A and B are considered the most relevant. Komori and Morimoto (1989) isolated trichomycin A using flash chromatography followed by HPLC, achieving a retention time of approximately 12 min. The isolated compound was obtained as a yellow powder. Its molecular formula (C₅₈H₈₄N₂O₁₈) and molecular weight (1096 u) were determined via fast atom bombardment (FAB) mass spectrometry. Solubility testing showed full solubility in DMSO, partial solubility in methanol and acetone, and insolubility in ethanol and water^29,31,32^. Trichomycin B, also isolated by HPLC, eluted at approximately 14 min and was likewise obtained as a yellow powder. It shares the same molecular formula and mass as trichomycin A, suggesting the presence of structural isomers^32^.

The constitution of trichomycin A was proposed by Hattori and colleagues in the 1960 s based on degradation studies^33^. The structure of trichomycin B was deduced by comparing the ¹³C NMR spectra of both compounds. While many signals overlapped, notable differences were observed at C4 and C12. In trichomycin B, the signals for C4 and C5 appeared at lower chemical shifts, whereas the C9 signal was significantly upfield compared to trichomycin A, suggesting that a hydroxyl group may be relocated from C5 to C9 in trichomycin B^31,32^. Importantly, no aromatic heptaene polyenes with an all-trans polyene chain are currently known, raising doubts about the accuracy of the 1990 structural proposals for trichomycins A and B.

In this study, we present an integrated re-assessment of the trichomycin complex focused on its two principal constituents. We analyze its composition using advanced analytical techniques, propose an effective method for the isolation of individual components, revise the 2D structures of the major constituents using two-dimensional NMR spectroscopy, and determine their full stereostructures by integrating NMR data with computational methods — including a generalized Karplus equation and metadynamics simulations.

## Results and discussion

### Analytical studies on trichomycin complex

**Fig. 1:**
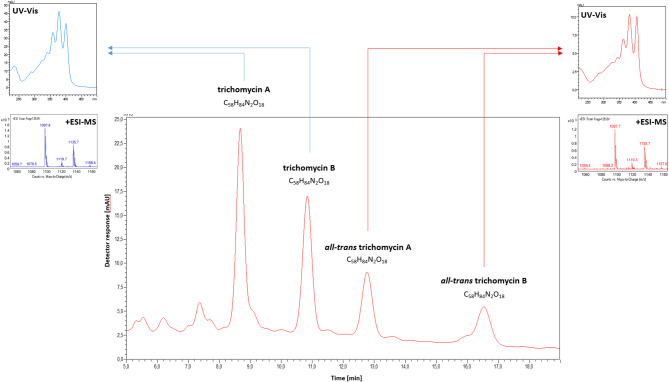
HPLC–DAD–ESIMS chromatogram of trichomycin complex. Chromatographic conditions: column Phenomenex Luna C18(2) 100 Å (150 mm × 4.6 mm, 5 μm). Mobile phase composition: 36% acetonitrile/64% ammonium acetate buffer (5.5 mmol, pH = 4.7), v/v; at a flow rate of 1 mL/min; inj. volume = 20 µL. Detection at 407 nm, room temperature.

Qualitative HPLC-DAD-ESIMS analysis (Fig. 1) revealed the presence of numerous compounds constituting the trichomycin antibiotic complex. Among them, four major constituents exhibited the highest relative abundances, eluting at retention times of t_R1_ = 8.7 min (32%), t_R2_ = 10.8 min (23%), t_R3_= 12.8 min (11%) and t_R4_ = 16.5 min (9%), respectively. The individual contributions of the remaining compounds did not exceed 4% and early-eluting species (t_R_< 6 min) are presumed to be degradation products of aromatic polyene macrolides, as suggested by their UV-Vis spectral profiles. Chromatographic integration was performed on the chromatogram recorded at λ₁ = 407 nm, which provided superior detection sensitivity for the compounds eluting at t_R3_ = 12.8 min and t_R4_ = 16.5 min.

UV-Vis absorption spectra collected for the first two major peaks (t_R1_ = 8.7 min and t_R2_ = 10.8 min) were characterized by three absorption maxima at λ_1_ = 360 nm, λ_2_ = 378 nm, and λ_3_ = 401 nm, with the band at 378 nm being the most intense (Fig. 1). This spectral pattern is characteristic of heptaene chromophores with *cis–trans* geometry^34,35^, suggesting that the native trichomycins possess a *cis–trans*-configured polyene system. This assumption is consistent with their classification as aromatic heptaene macrolides (AHMs) and their biogenetic origin — to date, no natural AHM with an *all-trans* chromophore has been identified^36^. Additional minor antibiotics within the trichomycin complex (e.g., t_R_ = 7.3 min) exhibited similar UV-Vis profiles indicative of *cis–trans* geometry, although their abundance was negligible and they will not be further discussed here. In contrast, the remaining two major peaks (t_R3_ = 12.8 min and t_R4_ = 16.5 min) displayed spectral features shifted bathochromically, with absorption maxima at λ_1_ = 364 nm, λ_2_ = 384 nm and λ_3_ = 407 nm, the latter being the most intense. This pattern is characteristic of amphotericin B and other polyene macrolides featuring an *all-trans* chromophore^36^.

Mass spectral analysis (positive ion mode) of all four principal peaks consistently revealed pseudomolecular ions at *m/z* [M + H]^+^ = 1097.7 (dominant), [M + K]^+^ = 1135.7 (less intense) and [M + Na]^+^ = 1119.7 (least intense), what strongly suggest that the four compounds are isomers of one another. Notably, the observed mass is corresponding to a monoisotopic molecular mass of C₅₈H₈₄N₂O₁₈ = 1096.6 u and matches literature data for trichomycins A and B, which are also structural isomers^31,32^. However, molecular mass and UV-Vis data alone were insufficient to assign the chromatographic peaks unambiguously to trichomycins A and B. To resolve this, a reference standard of trichomycin A was analyzed under identical HPLC-DAD-ESIMS conditions. The standard compound eluted at t_R1_ = 8.7, matching the retention time, UV-Vis profile and mass spectrum of the first major peak in the analyzed mixture (Figure S1). Accordingly, the t_R1_ = 8.7 peak was identified as trichomycin A, while the t_R2_ = 10.8 min peak was assigned to trichomycin B, an identification further supported by the NMR spectroscopic data presented below.

It is worth mentioning here, that in our earlier studies on other AHM complexes - candicidin (syn. levorin or ascosin) and partricin (syn. aureofacin), we demonstrated that these antibiotics undergo photochemical isomerization of their chromophores from *cis–trans* to *all-trans*, with the chromophore being the sole region affected by this structural rearrangement^10,37,38^. The resulting isomers were found to be stable in given conditions, and the transformation occurred under exposure to ambient light. This phenomenon may explain the overestimated number of reported components in many AHM complexes in the literature. It is therefore highly probable that the additional two major components in the trichomycin complex at the retention times t_R3_ = 12.8 min and t_R4_ = 16.5 min represent *all-trans* isomers of trichomycins A and B, respectively. This conclusion is supported by UV-Vis and MS profiles of these compounds, which were formed upon exposure of the native trichomycin complex to natural daylight.

Considering all of the above findings, we conclude that the native trichomycin complex consists primarily of two major antibiotics with the *cis-trans* chromophore— trichomycin A and trichomycin B — each comprising approximately 30–40% of the mixture, along with several minor components also with *cis–trans* chromophore geometry whose structures remain uncharacterized. These results shed new light on the actual number of bioactive constituents in the trichomycin complex and call into question previous reports describing the mixture as containing 6^29^, 10^27^ or even more than 16 components^28^. The apparent overestimation can likely be attributed to photochemically induced isomerization of the chromophores of native trichomycins.

### Preparative isolation of trichomycins A and B

In view of the fact that the stereostructure of both antibiotics remains undetermined and that these compounds are not commercially available, it was necessary to develop an effective isolation method. Since the chromatographic conditions optimized for analytical HPLC-DAD-ESIMS provided sufficient resolution, these parameters were adopted for model-scale separation (solvent system: 36% acetonitrile in 5.5 mmol ammonium acetate buffer, pH 4.7; column: Luna C18(2), 100 Å, 5 μm, 150 × 4.6 mm).

As a first step, concentration and volume overloading were determined. Due to the low solubility of the trichomycin complex in organic solvents and its negligible solubility in water, the mixture was dissolved in a DMSO–MeOH solvent system (1:3, v/v). DMSO was selected as the solvent providing the best solubility for AHM, including trichomycin, whereas MeOH was chosen as the co-solvent since these compounds show limited but acceptable solubility in lower alcohols.Increasing the proportion of DMSO beyond this ratio led to excessive mass transfer across the column, loss of resolution, and risk of precipitation of the antibiotics on the stationary phase under the applied chromatographic conditions due to significant solvent–system mismatch. Therefore a DMSO–MeOH (1:3) mixture provided sufficient solubility while maintaining compatibility with the chromatographic mobile phase, and was therefore chosen as the optimal vehicle for sample application. This approach yielded a maximum achievable concentration of 7 mg/mL, which is substantially lower than typically achieved in classical concentration overloading. At this concentration, the volume overloading was subsequently determined to be 100 µL. Preparative separation was performed on a 30 mm i.d. prep-HPLC column, resulting in a scaling factor x_prep_ of 42.5, as calculated using the Eq. 1 (see Methods Section). Based on this scaling factor, both eluent flow rate and injection volume were proportionally adjusted. Following initial preparative trials, it was found beneficial to reduce the acetonitrile content by 2% in order to improve peak separation between trichomycins A and B.

**Fig. 2:**
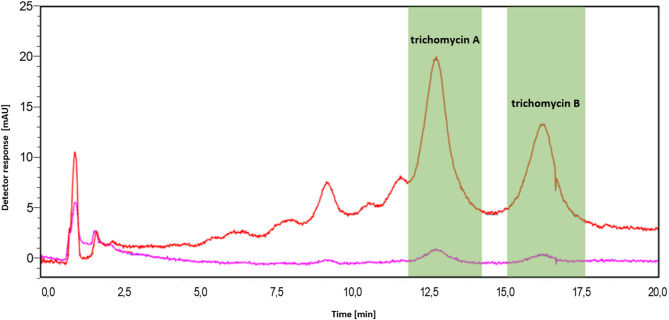
PrepHPLC-DAD chromatogram of trichomycin complex. The red chromatogram was obtained at 378 nm, while the magenta one was recorded at 254 nm. Chromatographic conditions: column Phenomenex Luna C18(2) 100 Å (150 mm × 30 mm, 5 μm). Mobile phase composition: 34% acetonitrile/66% ammonium acetate buffer (5.5 mmol, pH = 4.7), v/v; at a flow rate of 42.5 mL/min; inj. volume = 4.25 mL, sample conc.=7 mg/mL, room temperature.

The preparative-scale separation of the trichomycin complex is presented in Fig. 2. The fraction collection points corresponding to trichomycin A and subsequently trichomycin B were marked in green. The observed increase in retention times relative to the model scale resulted from reduced acetonitrile concentration in the mobile phase, as well as differences in instrumentation.

The collected fractions were subsequently converted into solid form using a method described in Methods Section. It is worth noting that, although two methods for separating the trichomycin complex had previously been proposed, none of them were suitable for preparative-scale applications^27,28^. Moreover, the prep-HPLC method developed later still required additional purification step to achieve the desired compound purity^29^.

In contrast, the method developed in the present study enabled the one-step isolation of both trichomycin A and trichomycin B in analytically pure form, without the need for further purification. However, it should be emphasized that this method—like other approaches employing preparative HPLC—is not the most suitable technique for isolating aromatic polyene macrolides. This is due to the low loading capacity of the chromatographic systems used and the phenomenon of irreversible adsorption of these antibiotics onto chromatographic columns (including reversed-phase HPLC), which results, on the one hand, in low purification efficiency and, on the other, in accelerated degradation of expensive preparative columns. To mitigate this phenomenon, various strategies are commonly employed, such as the use of additives in the mobile phase (e.g., acids or salts) and optimization of pH, temperature, or the elution gradient^39^. In the present study, after completing the separation of the trichomycin complex, the column was regenerated under isocratic elution conditions using 96% methanol in water at an elevated temperature of 45 °C, while maintaining the same flow rate as employed during the preparative run (42.5 mL/min). During this cleaning procedure, a blank injection of pure DMSO (4.25 mL) was introduced to promote complete desorption of residual analytes and to restore the column’s chromatographic performance.

Given the above-mentioned limitations, centrifugal partition chromatography (CPC) represents a particularly promising alternative for future applications, as this liquid–liquid technique completely eliminates the problem of irreversible adsorption by avoiding the use of a solid stationary phase. In addition to minimizing sample loss and column degradation, CPC offers several practical advantages, including mild operating conditions that preserve the integrity of labile natural products, straightforward solvent system optimization and excellent reproducibility between runs. Importantly, the technique allows for seamless scale-up from laboratory to pilot or industrial levels without changes to the separation principle, making it highly suitable for the preparative isolation of complex natural metabolites^40^. In our laboratory, ongoing work focuses on developing CPC-based protocols for the isolation of other aromatic polyene macrolide complexes, and the results of these studies will be presented in a forthcoming manuscript.

### 2D NMR studies on trichomycins A and B

The reconstruction of the spin systems and stereochemical frameworks of trichomycin A (Figure S4-S9) and B (Figure S10-S15) was accomplished using a standard repertoire of two-dimensional NMR experiments, including DQF-COSY, TOCSY, edited ¹H–¹³C HSQC, ¹H–¹³C HMBC, and ROESY spectra. This analytical strategy, previously employed for elucidating the stereostructures of various polyene macrolides—such as candicidin D^35^, A3^23^, and both partricin analogues^22^—proved equally effective in the present case. The two components of the trichomycin complex were separated, and individual sets of 2D NMR spectra were recorded for each compound.

The H1′/C1′ correlation in the HSQC spectrum (Figure S8, Figure S14) served as the starting point for reconstructing the spin system of the D-mycosamine residue. This signal was selected due to the distinctive chemical shift range of the acetal carbon (90–110 ppm), which is the only acetal-type carbon exhibiting methine character in these molecules. The observed chemical shifts of H1′ (δ_H1′A_ = 4.932 ppm, δ_H1′B_ = 4.929 ppm) and C1′ (δ_C1′A_ = 97.16 ppm, δ_C1′B_ = 97.24 ppm) in both trichomycins, along with the measured C1’/H1’ coupling constants (^1^J_H1’/C1’_ = 156 Hz in both cases) pointed to the presence of a β−1′-glycosidic bond, a finding further substantiated by vicinal coupling constants (³J_H, H_) within the sugar moiety and by characteristic dipolar interactions (ROEs).

The mycosamine–aglycone junction was elucidated based on HMBC (Figure S9, Figure S15) correlations between H1′/C21 and H21/C1′, and further supported by the ROE signal between H1′ and H21. The C21–C15 segments of both antibiotics were found to be structurally identical and consistent with literature data^31,32^. Given the established stereochemistry of D-mycosamine, this residue was employed as an internal chiral probe, in accordance with the methodology outlined in our previous works^10,21–23,35,37,41–43^. Dipolar couplings between probe and aglycone protons—specifically H2′/H19 and H2′/H20b—enabled the assignment of the absolute configuration at C21 as 21*R* in both compounds. This assignment, in conjunction with coupling constant analysis for H19/H20a, H18/H19, and H17/H18 (each approximately 10 Hz, indicative of diaxial orientation), closure of the six-membered C15–C19 ring (supported by the H19/C15 HMBC correlation), and a cascade of dipolar couplings involving H18/H20a, H19/H22, and H20b/H21/H23, allowed us to confidently establish the stereochemical configuration of the C15–C19 segment as 15*R*, 17*S*, 18*R*, and 19*S*.

The differentiation between trichomycin A and B was based on the structure of the C1–C14 polyol fragment. The flat structure of trichomycin A previously proposed in the literature^32^ was found to be inconsistent with the actual distribution of oxygen functionalities in this region. Hydroxyl groups were located at C13, C11, C9, and C5—rather than at C13, C11, C7, and C5, as earlier suggested—based on detailed analysis of 2D NMR spectra. The presence of a ketone group at C3 was confirmed and aligned with earlier reports^32^. In contrast, the flat structure of the C1–C14 segment in trichomycin B proved to be consistent with existing literature^31^. Based on vicinal coupling constants (Table S1) and sequential dipolar interactions among methine protons (H13/H11/H9 in trichomycin A, and H13/H11/H9/H7 in trichomycin B), along with ROE cascades H6b/H8b/H10b/H12b/H14b/H16b and H6a/H8a/H10a/H12a/H14a/H16a (observed in both compounds), the absolute configurations within the C6–C14 region were assigned as 9*S*, 11*S*, 13*S* for trichomycin A, and 7*S*, 9*S*, 11*S*, 13*S* for trichomycin B. Further analysis of the ROE sequence H9/H7b/H5 and vicinal coupling constants in the C4–C8 region enabled the assignment of the absolute configuration at C5 in trichomycin A as 5*R*.

**Fig. 3:**
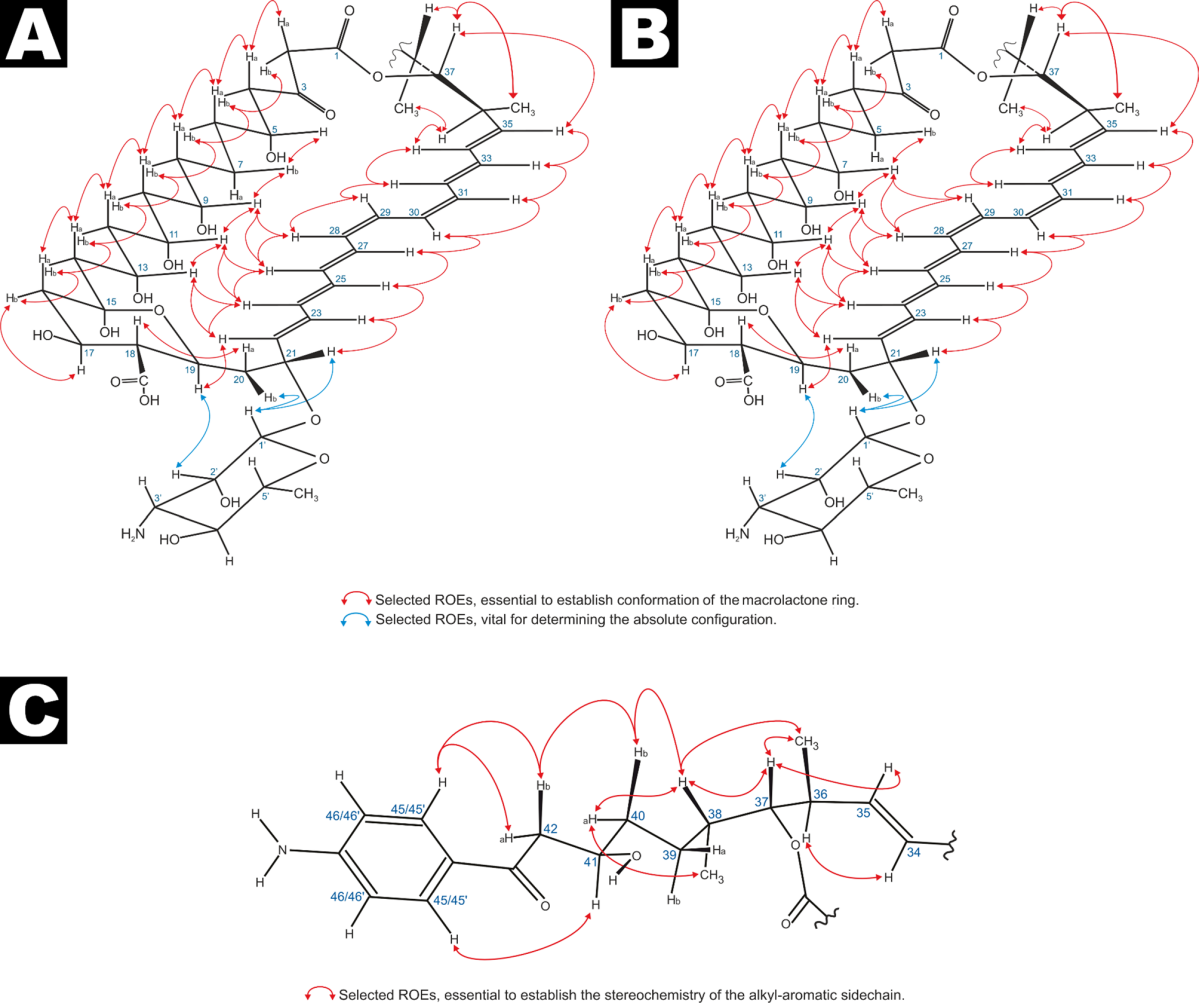
The stereostructure of trichomycin (**A**) and trichomycin (**B**) macrolactone rings, along with the stereostructure of alkyl-aromatic moiety of both antibiotics (**C**). Essential dipolar couplings are depicted as bidirectional arrows.

Naturally occurring representatives of aromatic heptaene macrolides with an all-trans chromophore geometry have not been described to date. Members of this class—also referred to as ‘aromatic analogues of amphotericin B’—are known to arise from their cis-trans chromophore counterparts through photochemical isomerization^10,37^. Thus, the chromophore geometry proposed for both trichomycins in earlier reports required re-evaluation. Indeed, based on vicinal coupling constants (J ~ 16 Hz for H22/H23, H24/H25, H26/H27, H32/H33, and H34/H35; J ~ 11 Hz for H28/H29 and H30/H31), and corroborated by ROE cascades H23/H25/H27/H30/H31/H33/H35 and H22/H24/H26/H28/H29/H32/H34 (Fig. 3, Table S1), the C22–C35 segment was assigned the configuration: 22*E*, 24*E*, 26*E*, 28*Z*, 30*Z*, 32*E*, 34*E*.

The structure of the aromatic side chain was consistent with existing reports^31,32^. The remaining unassigned stereocenters—C36, C37, C38, and C41—were resolved using a combination of ROE correlations (H34/H36, H35/H37, H36/Me38, H38/Me36) and vicinal coupling constants (³J_H36/H37_ = 10.5 Hz; ³J_H37/H38_ = 2.3 Hz). In particular, the ROE between H36 and H34 allowed determination of the relative configuration at C36 with respect to C37. A comprehensive analysis of all proton interactions, together with the observation that only one enantiomer of the C36–C38 fragment allows for successful macrolactone ring closure—while preserving both the coupling constants of the polyol region and the full ROE cascade from C2 to C14, as well as dipolar interactions between the heptaene chromophore and this fragment (Figure S21 and S22)—led to the assignment of configurations as 36*S*, 37*R*, and 38*S*.

Due to the absence of a methyl group at C40 and the overlap of the H40a/H40b resonances, it was not possible to differentiate these protons based on their couplings to H41. It is known, however, that one of them couples to H41 with a vicinal coupling constant of ~ 10.5 Hz. This implies that the C40–C41 bond has conformational preferences; however, the superposition of the H40a/H40b resonances effectively prevents their discrimination, and the ~ 10.5 Hz coupling cannot be unambiguously assigned to either proton. As a consequence, stereostructural considerations cannot be drawn directly from H40/H41 correlations. Nonetheless, ³J_H41/H42a_ and ³J_H41/H42b_ were measurable in both trichomycins, with values of ~ 3 Hz and ~ 8 Hz, respectively (see Table 1). Taken together, these findings point to restricted rotation about the C40–C41 and C41–C42 bonds, plausibly reflecting an intramolecular hydrogen bond between 41-OH and the carbonyl at C43. Consistent with this interpretation, ROESY spectra show the H41/H42b cross-peak to be ~ 25% more intense than H41/H42a, indicating a shorter interproton distance and supporting conformational rigidity in this region.

**Table 1:** .

	Coupling protons [i, j]	Measured ^3^*J*_i/j_ [Hz]	Calculated ^3^*Ĵ*_i/j_ [Hz]	
			41*R*	41*S*
Trichomycin A	H40a*, H41	10.5	2.59 ± 2.46 [2.43–2.75]	9.66 ± 3.64 [9.19–10.13]
	H40b*, H41		**9.62 ± 3.96 [8.93–10.31]**	3.92 ± 2.40 [3.73–4.11]
	H41, H42a	3.2	**4.14 ± 3.41 [3.73–4.55]**	9.46 ± 3.83 [9.06–9.86]
	H41, H42b	8.2	**9.35 ± 3.16 [8.95–9.75]**	3.26 ± 3.50 [3.02–3.49]
Trichomycin B	H40a*, H41	10.5	1.85 ± 1.42 [1.77–1.92]	9.66 ± 3.73 [9.26–10.06]
	H40b*, H41		**9.92 ± 2.48 [9.60–10.27]**	3.50 ± 3.65 [3.13–3.86]
	H41, H42a	3.3	**2.85 ± 2.23 [2.72–2.98]**	8.56 ± 3.20 [8.15–8.96]
	H41, H42b	8.1	**10.07 ± 2.17 [9.94–10.19]**	3.41 ± 2.34 [3.16–3.66]

Absolute configuration at C41 could not be conclusively established based on NMR data alone, yet comparison of experimentally measured coupling constants for the C40–C42 fragment with theoretical values predicted from molecular dynamics simulations for both 41*R* and 41*S* epimers—interpreted via the Haasnoot–de Leeuw–Altona modification of the Karplus equation (Eq. 2)^44^—allowed a meaningful inference. Additionally, the distances between H41 and H42a/b were related to the observed ROE intensities. A high-resolution spatial analysis of the chemical environment of protons H42a and H42b — incorporating the conformational restriction around the C40–C41 and C41–C42 bonds inferred from the measured vicinal coupling constants and the results of metadynamics simulations (Figure S18) — demonstrated that proton H42b is expected to resonate at a higher chemical shift in both the 41*R* and 41*S* epimers. Assuming this is correct, Table 1 shows that while one of the H40ab/H41 couplings is consistent with both epimers, the values of ³J_H41/H42a_, ³J_H41/H42b_, and the corresponding interproton distances are more consistent with the 41*R* epimers of trichomycin A and B. For the 41*S* epimers, results are inverted, and agreement would only be obtained by reassigning the diastereotopic protons H42a and H42b (Figure S17). Given that antibiotics in this family are biosynthesized through conserved pathways across numerous *Streptomyces* strains and species^45^, and that the 41*R* configuration has been assigned to other AHM members^22,23,35,37^, we propose the absolute configuration at C41 in trichomycin A and B as 41*R*.

However, because we cannot fully substantiate the premise that H42b resonates at a higher chemical shift in both epimers—and chemical-shift predictors are not decisive—and because, if this premise were incorrect, the 41*R*/41*S* differences in calculated vicinal couplings (Table 1) lie within methodological uncertainty, the 41*R* assignment should be regarded as provisional rather than definitive. Accordingly, we treat it as a working hypothesis consistent with the available indirect evidence. Conclusive validation will require orthogonal methods (e.g., X-ray crystallography or stereoselective synthesis/derivatization with known configuration).

## Conclusions

The structural investigations presented in this study have demonstrated that trichomycin belongs to the partricins (*syn.* aureofacins)^10^ subclass of aromatic heptaene macrolides. This group is characterized by the presence of two cis-configured double bonds at positions 28*Z* and 30*Z*, an alkyl–aromatic side chain lacking a methyl substituent at position C40, and a β−1′-glycosidically linked sugar moiety, identified as D-mycosamine. In contrast, the presence of a C40 methyl group—as observed in candicidin analogues^21,23,35,42^—shifts the location of the two cis double bonds to positions 26*Z* and 28*Z*. This relationship between the presence or absence of the Me40 substituent and the ‘bend point’ of the chromophore appears to be reproducible and consistent across all members of this antibiotic class^21,22,46^, suggesting that it is inherently linked to the molecular mechanism of biological activity, which remains incompletely understood and is still under active investigation.

**Fig. 4:**
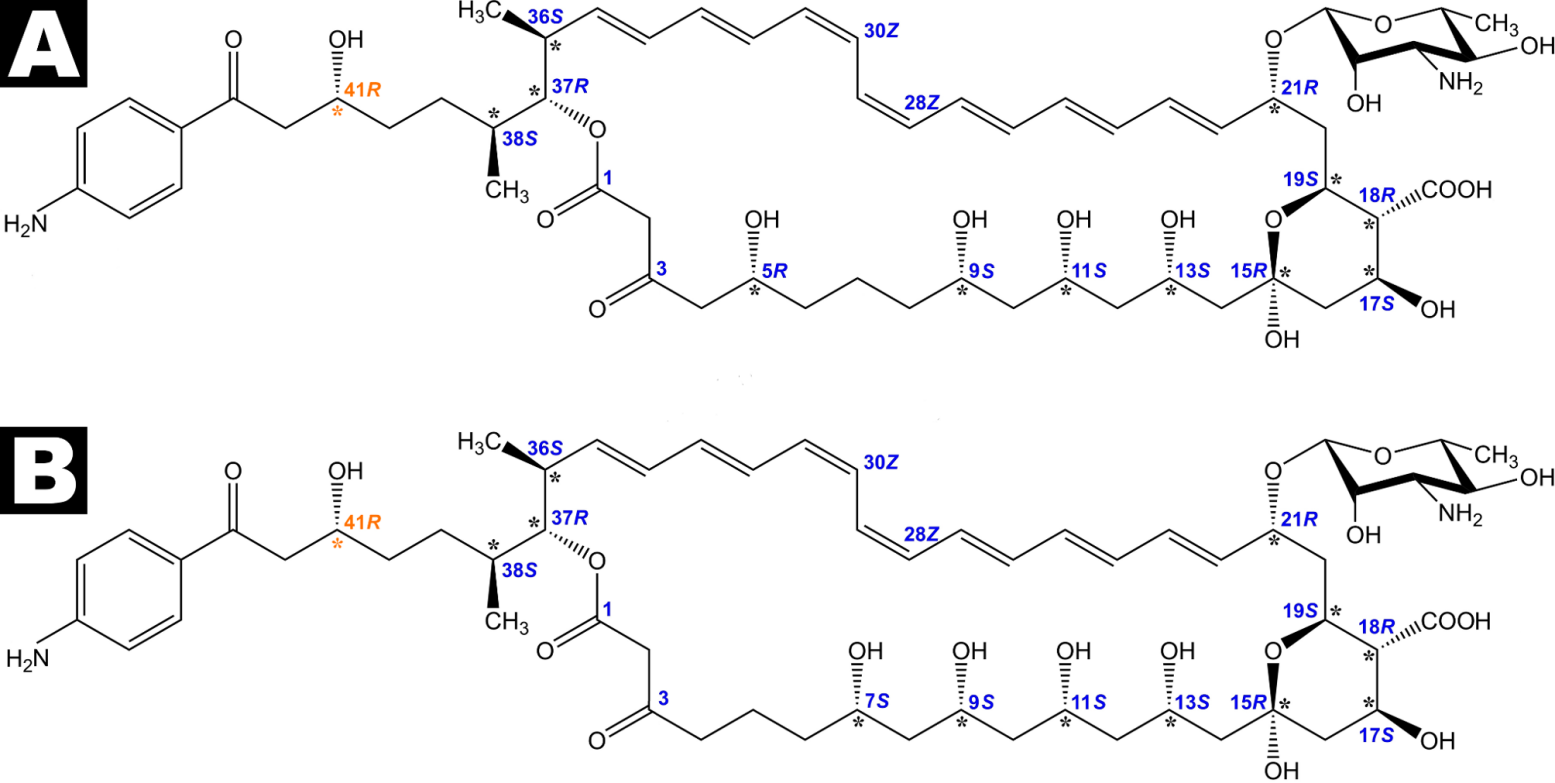
Revised structures of trichomycins (**A**) and (**B**) with full stereochemical assignments. The absolute configuration at C41 (highlighted in orange) is proposed and should be regarded as tentative, not definitive.

Based on a combination of 2D NMR experiments and molecular modeling calculations using a generalized Karplus equation, the absolute configuration of trichomycin A was established as: 5*R*, 9*S*, 11*S*, 13*S*, 15*R*, 17*S*, 18*R*, 19*S*, 21*R*, 36*S*, 37*R*, 38*S*, with 41*R* proposed (Fig. 4A). In the case of trichomycin B, the absolute configuration was determined as: 7*S*, 9*S*, 11*S*, 13*S*, 15*R*, 17*S*, 18*R*, 19*S*, 21*R*, 36*S*, 37*R*, 38*S*, with 41*R* likewise proposed (Fig. 4B). The chromophore geometry for both trichomycins was established as: 22*E*, 24*E*, 26*E*, 28*Z*, 30*Z*, 32*E*, 34*E*. Compared to studies published in the early 1990s^31,32^, this work not only defines the complete stereochemistry of both compounds, but also revises the hydroxylation pattern in the polyol region of trichomycin A.

While the 41*R* absolute configuration may appear to contradict the 41*S* configuration previously assigned to candicidin D^35^ and A3^23^, this apparent discrepancy arises from differences in CIP priority rules, owing to the presence of a C40 methyl group in candicidin. From a structural standpoint, the three-dimensional geometry of this fragment is essentially identical in both antibiotics — aside from the presence or absence of the Me40 substituent (Fig. 4).

This study offers new insights into the structural complexity of the Trichomycin complex. Discrepancies in the literature regarding the true number of components in the complex may be explained by photochemical isomerization of native *cis–trans* trichomycins into their *all-trans* isomers, effectively doubling the number of observed components. Moreover, the prep-HPLC isolation protocol developed herein enables single-step purification of trichomycins A and B in analytically pure form, eliminating the need for subsequent purification steps, as none of them is commercially available.

The complete analytical and structural characterization of trichomycin presented in this study, together with the development of an efficient isolation protocol for its individual components, lays the foundation for the rational molecular modification of this compound aimed at improving its pharmacological properties. These may include photochemical isomerization^10,37^, targeted modification of the polar headgroup (as demonstrated in the recent work by Burke et al.), or other derivatization strategies^6–8,19,47,48^. Additionally, by establishing the stereochemistry of trichomycin B, we have defined a molecule that displays close structural similarity to a representative of another AHM subclass— the candicidins^10,35^, namely candicidin D. Apart from the oxidation state at C7, the only structural differences between candicidin D and trichomycin B are the presence or absence of a methyl group at C40 and the location of the chromophore “kink” (26*Z*,28*Z* in candicidin D vs. 28*Z*,30*Z* in trichomycin B). This provides the strongest framework to date for probing how (i) the location of the chromophore kink and (ii) the rigidity of the alkyl–aromatic side chain—largely governed by the C40 methyl group—together modulate the biological activity of aromatic heptaene macrolides. Moreover, by photochemically isomerizing trichomycin B into its *all-trans* isoform in future work, we can remove chromophore geometry as a confounding variable and directly compare the activities of the iso-trichomycin B with iso-candicidin D. Such a design will isolate the contribution of side-chain dynamics to AHM bioactivity and help clarify the role of this structural fragment in the molecular mechanism of action of this class of antibiotics.

The quest for a successor to amphotericin B—the long-standing *gold standard* in the treatment of systemic fungal infections—remains one of the most urgent challenges in antifungal therapy. Developing a molecule that matches or surpasses its efficacy, while providing a superior selectivity index and improved pharmacokinetic profile, constitutes a major objective in advancing global public health.

## Methods

### Analytical studies

#### Antibiotics used in the study

Preparations of the trichomycin complex, obtained by prior fermentation of *Streptomyces hachijoensis* and the reference standard of trichomycin A were obtained from the collection of the Department of Pharmaceutical Technology and Biochemistry, Gdańsk University of Technology.

#### Analytical HPLC-ESI-MS studies of trichomycin samples

Qualitative analysis of the trichomycin complex sample, trichomycin A reference standard and isolated trichomycin A and B was performed by an analytical platform—the HPLC-ESI-MS/MS (QQQ) chromatograph 1260 Infinity II Series (Agilent Technologies, Santa Clara, CA, USA) composed of a binary pump, a degasser, an autosampler, a thermostat, a DAD detector and mass spectrometer (G6470A). The following chromatographic conditions were applied in the study: flow rate of 1 mL/min, temperature of 21 °C, injection volume of 20 µL, the UV signals: 378, 407, 254 and 320 nm and the run time of 40 min. The gradient of acetonitrile (solvent B) and ammonium acetate buffer (5.5 mmol, pH = 4.7) was used in the following program: 0 min—36% B, 20 min—36% B, 25 min—95% B, 30 min—95% B, 35 min 36% B, 40—36% B. The chromatographic separation was performed on a Phenomenex Luna C18(2) 100 Å column (150 mm × 4.6. mm; dp = 5 μm) produced by Phenomenex (Torrance, CA, USA).

Freshly calibrated and tuned mass spectrometer was operated within the m/z range of 800–1200 u, in the further described settings: gas temperature of 300 °C, sheath gas temperature of 250 °C, gas flow of 5 L/min and sheath gas flows of 10 L/min, nebulizer pressure of 60 psig, capillary voltage of 3000 V, fragmentor voltage of 135 V. Mass Hunter Workstation Software (v. B.08.00) was used to record and process the collected data.

### Prep-HPLC isolation of trichomycins A i B

Preparative isolation of trichomycins A and B was performed on a Teledyne ISCO apparatus ACCQPrep HP125 equipped with DAD detector and fraction collector (Teledyne ISCO, Lincoln, NE, USA). The chromatographic conditions were as follows: column Phenomenex Luna C18(2) 100 Å (150 mm × 30 mm; dp = 5 μm), mobile phase composition: 34% acetonitrile/66% ammonium acetate buffer (5.5 mmol, pH = 4.7), v/v; flow rate 42.5 mL min − 1; detection at 378 nm; room temperature. Both the flow rate and the injection volume were scaled using the x_prep_ scaling factor of 42.5, calculated based on Eq. 1.

1 $$x_{prep}=\frac{d^2_2}{d^2_1}=\frac{30^2}{4.6^2}=42.5$$

where:

*d₂* – internal diameter of the preparative column,

*d₁* – internal diameter of the analytical (model) column.

For the separation, 30 mg of trichomycin complex sample was dissolved in 1 mL of DMSO and then dissolved with MeOH to the final volume of 4.25 mL and filtered before injection into the column. Isocratic elution was performed for 25 min, followed by a washing step of a column. Trichomycin A eluted from the column within 12–14.5 min, while trichomycin B eluted within 15.5–17.5 min of process giving ca. 106 mL and 85 ml of collected fractions, respectively. Next, trichomycin A and B fractions were concentrated under reduced pressure (5–7 mbar) at temperature not exceeding 35 °C, removing acetonitrile. Both antibiotics were then extracted from the remaining aqueous layer (containing ammonium acetate) using 50 mL of n-butanol. The obtained organic phases were desalted by washing with 20 mL of ultrapure water (2 × 10 mL) and further concentrated to a volume of approximately 5 mL. Next, the antibiotics were precipitated from the butanol solution using 3 mL of diethyl ether and centrifuged in a preparative centrifuge for 5 min at 2400 rpm. The supernatants were then discarded, and the antibiotic-containing precipitates was washed with 3 mL of diethyl ether and centrifuged again under the previously described conditions. Finally, the precipitates containing trichomycin A and trichomycin B were dried in a vacuum desiccator for 24 h, protected from light. The final yield was 3.3 mg for trichomycin A (Figure S2) and 2.8 mg for trichomycin B (Figure S3), both with the purity of ca. 86% (HPLC-DAD analysis).

### Optical rotation

The optical rotation of trichomycin A and trichomycin B was measured with an Autopol II automatic polarimeter (Rudolph Research, Flanders, NJ, USA) in dimethylformamide (DMF) with a sample concentration of ~ 5 mg/mL at λ = 589 nm. The recorded optical rotation value was [ɑ]_D_^20^ +127.8° for trichomycin A and [ɑ]_D_^20^ +135.5° for trichomycin B, respectively.

### NMR experimental details

NMR spectra of both trichomycins were recorded on a Bruker Avance III HD 700 MHz spectrometer equipped with a QCI CryoProbe. All measurements were performed at ambient temperature in a pyridine-d₅/methanol-d₄ (9:1, v/v) solvent system, with a sample concentration of ~ 12 mg/mL. Chemical shifts (δ) are reported in parts per million (ppm), referenced to the residual proton signal of pyridine-d₅ at δ_H_ = 7.19 ppm.

One-dimensional ¹H NMR spectra (Figure S4, Figure S10) were acquired with a digital resolution of 0.5 Hz, and a 90° pulse length of 7.2 µs. Two-dimensional ¹H–¹H spectra were recorded in phase-sensitive mode with a spectral width of 7716 Hz. All 1D and 2D spectra were acquired with a recycle delay d₁ = 2.0 s. No quantitative NMR analyses were performed, and integrals were not used for structural deductions. The set of experiments (DQF-COSY, TOCSY, HSQC, HMBC, ROESY) provides through-bond and through-space connectivities that are independent of absolute T₁ values; the chosen d₁ prevents differential saturation from affecting peak detection and assignment.

DQF-COSY spectra (Figure S5, Figure S11) were collected in a 4096 × 512 matrix, with 32 scans per increment, and processed as 4 K × 2 K data matrices.

TOCSY spectra (Figure S6, Figure S12) were acquired using a mixing time of 60 ms, with a 2048 × 512 matrix, 8 scans per increment, and processed as 2 K × 1 K.

ROESY spectra (Figure S7, Figure S13) were recorded with a mixing time of 350 ms, in a 2048 × 512 matrix, using 48 scans per increment, and processed as 2 K × 1 K matrices.

Two-dimensional ¹H–¹³C HSQC and HMBC (Figure S9, Figure S15) experiments were performed using gradient-selected pulse sequences.

HSQC and edited-HSQC (Figure S8, Figure S14) spectra were acquired in phase-sensitive mode with the ¹J_C, H_ coupling constant set to 140 Hz. Spectral widths were 7716 Hz (¹H) and 29,177 Hz (¹³C). Data were collected in a 2048 × 256 matrix, with 56 scans per increment, and processed as 2 K × 1 K matrices.

HMBC spectra were acquired in absolute value mode, with long-range ^n^J_C, H_ coupling constant set to 9 Hz. Spectral widths were 7716 Hz (¹H) and 40,515 Hz (¹³C). The data were collected in a 2048 × 256 matrix, with 184 scans per increment, and processed as 2 K × 1 K.

### Molecular modelling studies

#### Molecular dynamics

Molecular models of both epimeres for trichomycin A and B were built in an open-source molecular builder and visualization tool - Avogadro^49^. Parameters for the molecular models of all versions of antibiotics were derived from the CHARMM36 Generalized Force Field^50^. The molecular geometries were optimized at the DFT level of theory (MN12SX/6-31G*) using Gaussian 16, and all optimizations converged within Gaussian default thresholds (see Supplementary Information for convergence data (Table S2) and optimized structures (Figure S16, Table S3)). Partial atomic charges for force field parametrization were calculated using electrostatic potential (ESP) fitting at the MP2/6-31G* level of theory and dihedral definitions were refined using the ForceField Tool Kit (ffTK) within VMD 1.9.2^51,52^.Four systems were prepared: trichomycin A with R configuration at carbon 41, trichomycin A with *S* configuration at carbon 41, trichomycin B with *R* configuration, and trichomycin B with *S* configuration. Each antibiotic was solvated in a cubic box of pyridine, containing 1523, 1521, 1527, and 1527 pyridine molecules, respectively. Pyridine was selected as the solvent to match the NMR experimental conditions, with its parameters sourced from the CHARMM36 Generalized Force Field^50^.

Following energy minimization, each system underwent a two-stage equilibration with positional restraints applied to the heavy atoms of the antibiotic. The first stage consisted of a 100 ps NVT simulation, followed by a 200 ps NPT simulation. Both stages employed a 2 fs time step, the leapfrog integrator, and a temperature of 300 K maintained by the velocity-rescaling thermostat (τ_t_ = 0.1 ps). In the NPT stage, pressure was controlled at 1 bar using a weak-coupling barostat (τ_p_ = 2 ps). All bonds involving hydrogen atoms in the system were constrained using the P-LINCS algorithm.

After equilibration, positional restraints were removed, and a 500 ns production run was conducted in the NPT ensemble. The temperature was maintained at 300 K with the velocity-rescaling thermostat (τ_t_ = 0.1 ps), and the pressure was held at 1 bar using the C-rescale barostat (τ_p_ = 2 ps). Electrostatic interactions were calculated using the Particle Mesh Ewald (PME) method with a 1.0 nm cutoff and an approximate grid spacing of 0.1 nm. Van der Waals forces were modeled with a 1.0 nm Lennard-Jones cutoff^53,54^. All simulations were performed using GROMACS 2024.2^55,56^.

#### Haasnoot-de leeuw-altona (HLA) equation

Upon completion of all molecular dynamics (MD) simulations (**tryAR**, **tryAS**, **tryBR**, and **tryBS**), a representative statistical ensemble comprising 2000 frames per conformer was extracted from the resulting 500 ns trajectories. For each frame, the vicinal ³J coupling constants — ³J_H40a/H41_, ³J_H40b/H41_, ³J_H41/H42a_, and ³J_H41/H42b_ — were calculated using the HLA equation (Eq. 2):

2 $$^3J=P_1{cos}^2\phi+P_2{cos}\phi+P_3+\sum\Delta{\chi}_i[P_4+P_5{cos}^2(\varepsilon_i\phi+P_6\mid\Delta\chi_i\mid)]$$

The resulting 2000 coupling values for each dihedral of interest were averaged across the ensemble, yielding the mean ensemble coupling constants denoted as ³Ĵ_H40a/H41_, ³Ĵ_H40b/H41_, ³Ĵ_H41/H42a_, and ³Ĵ_H41/H42b_, as presented in Table 1.

For all calculations, the following empirical HLA parameters were used: P₁ = 14.64, P₂ = − 0.78, P₃ = 0.58, P₄ = 0.34, P₅ = − 2.31, and P₆ = 18.4, in accordance with values implemented in MestReJ software^57^.

The ξ_i_ parameters, accounting for the stereoelectronic orientation of substituents, were set to + 1 or − 1, depending on the local geometry of each S_i_ substituent (Table S4). The Δχ_i_ parameters, reflecting the electronic/inductive properties of substituents, were also taken from MestReJ and varied according to substituent identity (Table S4).

#### Estimation of confidence intervals for MD-derived ³J couplings

For each epimer and dihedral, instantaneous ³J values were computed for every MD frame using the generalized Haasnoot–de Leeuw–Altona (HLA) equation. Ensemble results are reported as mean ± SD (dispersion due to conformational heterogeneity). To account for temporal correlation between frames, we estimated the integrated autocorrelation time τ_int_ of each ³J time series and derived the effective sample size N_eff_ = N/τ_int_. 95% confidence intervals (CI, Eq. 3) for the mean were then computed as:

3 $$\overline{J}\pm{t}_{0.975,N}{_{eff}}^-1\frac{s}{\sqrt{N_{eff}}}$$

where $\:\underset{\_}{J}$ is the sample mean, *s* is the standard deviation, and *t* is the Student’s *t*-quantile. Autocorrelation functions *ρ(k)* were summed up to the first non-positive lag (“Sokal” truncation) to obtain $\:{\tau\:}_{int}=1+2{\sum\:}_{k=1}^{K}\rho\:\left(k\right)$. We capped $\:{N}_{eff}\le\:N$. Scripts used for these analyses are available upon request.

#### Metadynamics

The free energy profiles (Figure S18) of the H40a/H41, H40b/H41, H42a/H41, H42b/H41 dihedral angles of each diastereoisomer of trichomycin A and B were obtained with the use of metadynamics^58–60^. For the system prepared as stated above, 16 well–tempered metadynamics runs were carried out along the reaction coordinate set as H40a-C40-C41-H41, H40b-C40-C41-H41, H42a-C42-C41-H41, H42b-C42-C41-H41 dihedral angles with a bias factor of 4 and simulation length of 500 ns. The history dependent bias was deposited every 5 000 steps as Gaussians with height of 0.01 kcal/mol and sigma equal to 0.01 kcal/mol. The temperature was set to 300 K. Convergence of the simulations was validated through complementary evaluations of time-dependent RMSD and block-averaged free energy errors (Figures S19–S20). The rest of the simulation parameters were set as described in the previous section.

## Supplementary Information

Below is the link to the electronic supplementary material.

Supplementary Material 1

## Data Availability

Most of the data generated or analyzed during this study are included in this published article (and its Supplementary Data files). The full 2D NMR spectra and MD trajectories generated and analyzed during the current study are available from the corresponding author on reasonable request.
