# Supplementary material for: Isolation, structure revision and stereochemistry of trichomycins A and B, aromatic analogues of amphotericin B
Source: Sci Rep. 2025 Dec 29;15:44862. doi: 10.1038/s41598-025-28626-x (PMC12748807; doi:10.1038/s41598-025-28626-x)
Supplement: Supplementary file 1 — Supplementary Material 1 [file 41598_2025_28626_MOESM1_ESM.pdf]

Isolation, Structure Revision and Stereochemistry of Trichomycins A and B, Aromatic Analogues of Amphotericin B

Tomasz Laskowski[1], Filip Anaszewicz[1], Julia Borzyszkowska-Bukowska[1], Julia Pakuła[1], Małgorzata Michałowska[1], Julia Bublewska[1], Dorota Gudanis-Sobocińska[2], Katarzyna Kozłowska-Tylingo[1], Paweł Szczeblewski[1,*]

[1] Department of Pharmaceutical Technology and Biochemistry and BioTechMed Centre, Chemical Faculty, Gdansk University of Technology, Gabriela Narutowicza Str. 11/12, 80-233, Gdańsk, Poland

[2] Department of Biomolecular NMR, Institute of Bioorganic Chemistry Polish Academy of Sciences, Zygmunta Noskowskiego Str. 12/14, 61-704, Poznań, Poland

* Correspondence should be addressed to P. Sz. (pawel.szczeblewski@pg.edu.pl), Gabriela Narutowicza Str. 11/12, 80-233 Gdańsk, phone number: +4858 3472079, fax: +4858 347 11 44.

SUPPLEMENTARY INFORMATION

Table of content

Tables

Table S1. 1H and 13C NMR data for trichomycin A and B. 6
Table S2 Convergence data for QM calculations for all studied molecules............................ 23
Table S3 The XYZ coordinates for both enantiomers of Trichomycin A and B. 24
Table S4. Parameters for HLA equation used for trichomycin A and B. ‘S1-S4’ stand for ‘substituents 1-4’. 35

Figures

Figure S1. Superimposed HPLC–DAD chromatograms of trichomycin complex (red line) and trichomycin A reference standard (blue line). Chromatographic conditions: column Phenomenex Luna C18(2) 100 Å (150 mm × 4.6 mm, 5 μm). Mobile phase composition: 36% acetonitrile/64% ammonium acetate buffer (5.5 mmol, pH = 4.7), v/v; at a flow rate of 1 mL/min; inj. volume = 20 μL. Detection at 407 nm, room temperature. 3
Figure S2 The HPLC-DAD-ESIMS chromatogram of isolated Trichomycin A sample. Chromatographic conditions: column Phenomenex Luna C18(2) 100 Å (150 mm × 4.6 mm, 5 μm). Mobile phase composition: 36% acetonitrile/64% ammonium acetate buffer (5.5 mmol, pH = 4.7), v/v; at a flow rate of 1 mL/min; inj. volume = 20 μL. Detection at 407 nm, room temperature. 4
Figure S3 The HPLC-DAD-ESIMS chromatogram of isolated Trichomycin B sample. Chromatographic conditions: column Phenomenex Luna C18(2) 100 Å (150 mm × 4.6 mm, 5 μm). Mobile phase composition: 36% acetonitrile/64% ammonium acetate buffer (5.5 mmol, pH = 4.7), v/v; at a flow rate of 1 mL/min; inj. volume = 20 μL. Detection at 407 nm, room temperature. 5
Figure S4. 1H NMR spectrum of trichomycin A 11
Figure S5. DQF-COSY spectrum of trichomycin A. 12
Figure S6. TOCSY spectrum of trichomycin A. Spin-lock = 60 ms. 13
Figure S7. ROESY spectrum of trichomycin A. Mix time = 350 ms. 14
Figure S8. Edited-HSQC spectrum of trichomycin A.. 15
Figure S9. HMBC spectrum of trichomycin A. 16
Figure S10. 1H NMR spectrum of trichomycin B.. 17
Figure S11. DQF-COSY spectrum of trichomycin B. 18
Figure S12. TOCSY spectrum of trichomycin B. Spin-lock = 60 ms. 19
Figure S13. ROESY spectrum of trichomycin B. Mix time = 350 ms. 20
Figure S14. Edited-HSQC spectrum of trichomycin B.. 21
Figure S15. HMBC spectrum of trichomycin B. 22
Figure S16 3D structures for both enantiomers of trichomycin A and B. 34
Figure S17. Distribution of the four dihedral angles during the 500 ns equilibrium MD simulations of the both possible trichomycin A enantiomers (left), distribution of the four dihedral angles monitored of the both possible trichomycin B enantiomers (right). 36
Figure S18. Free energy profiles for all monitored dihedral angles for both enantiomers of trichomycin A (left) and trichomycin B (right). 36
Figure S19 Time-dependent RMSD plots for both enantiomers of trichomycin A (upper) and trichomycin B (lower). 37

Figure S20 Block-averaged free energy errors for both enantiomers of trichomycin A (upper) and trichomycin B (lower). 38
Figure S21 C9-C15 hydrogen bonding network of Trichomycin A. 38
Figure S22 Trichomycin A with absolute configuration reversed at C36 and C37. 39

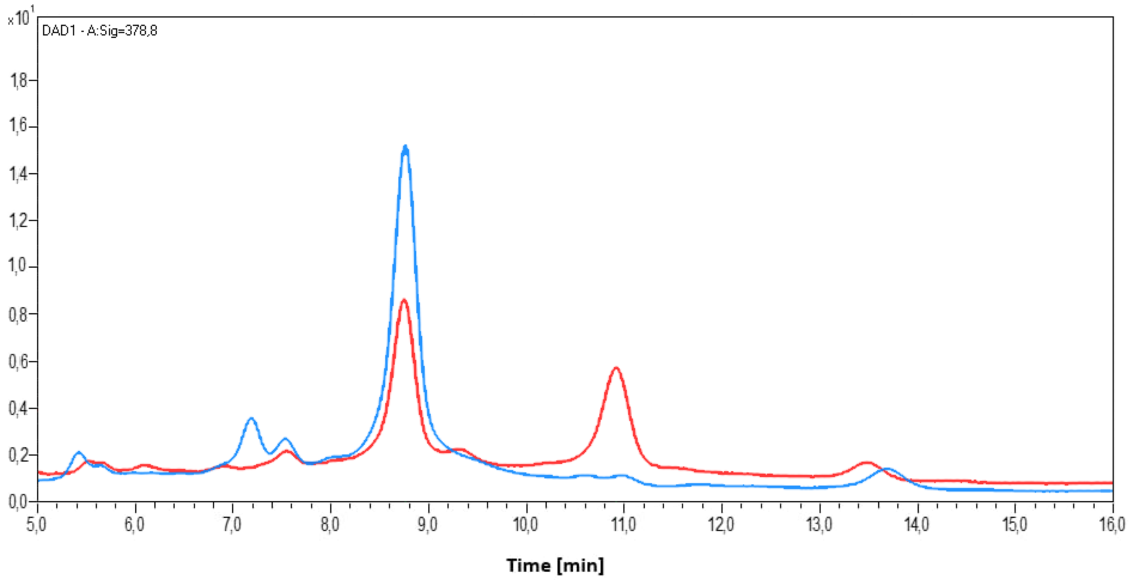

Figure S1. Superimposed HPLC–DAD chromatograms of trichomycin complex (red line) and trichomycin A reference standard (blue line). Chromatographic conditions: column Phenomenex Luna C18(2) 100 Å (150 mm × 4.6 mm, 5 μm). Mobile phase composition: 36% acetonitrile/64% ammonium acetate buffer (5.5 mmol, pH = 4.7), v/v; at a flow rate of 1 mL/min; inj. volume = 20 μL. Detection at 407 nm, room temperature.

Trichomycin A

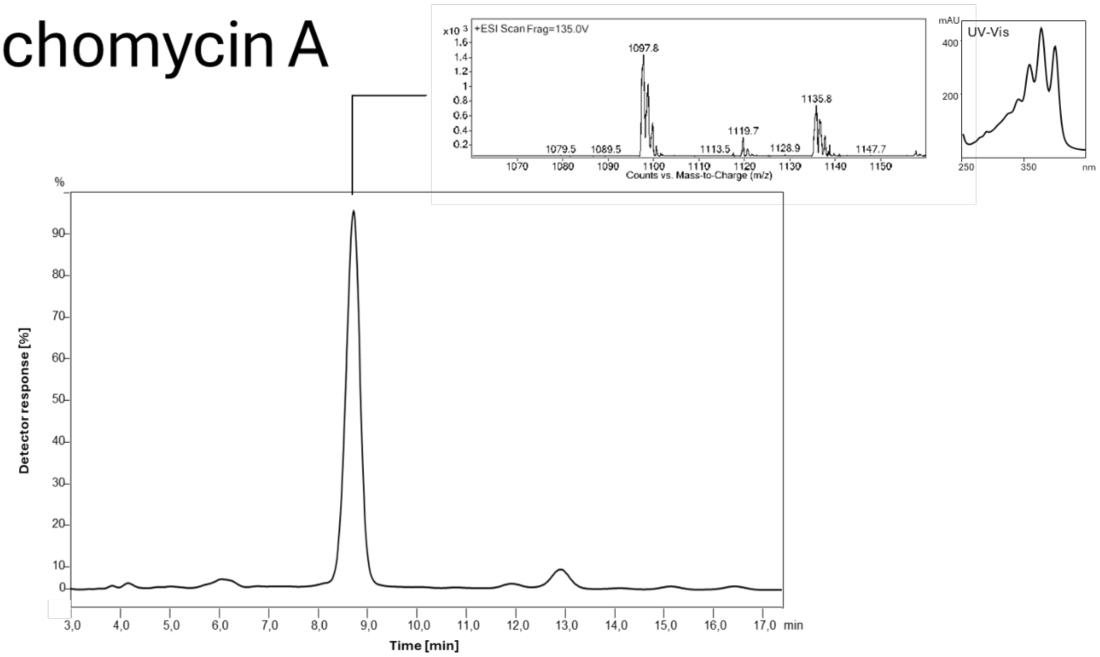

Figure S2 The HPLC-DAD-ESIMS chromatogram of isolated Trichomycin A sample. Chromatographic conditions: column Phenomenex Luna C18(2) 100 Å (150 mm × 4.6 mm, 5 µm). Mobile phase composition: 36% acetonitrile/64% ammonium acetate buffer (5.5 mmol, pH = 4.7), v/v; at a flow rate of 1 mL/min; inj. volume = 20 µL. Detection at 407 nm, room temperature.

Trichomycin B

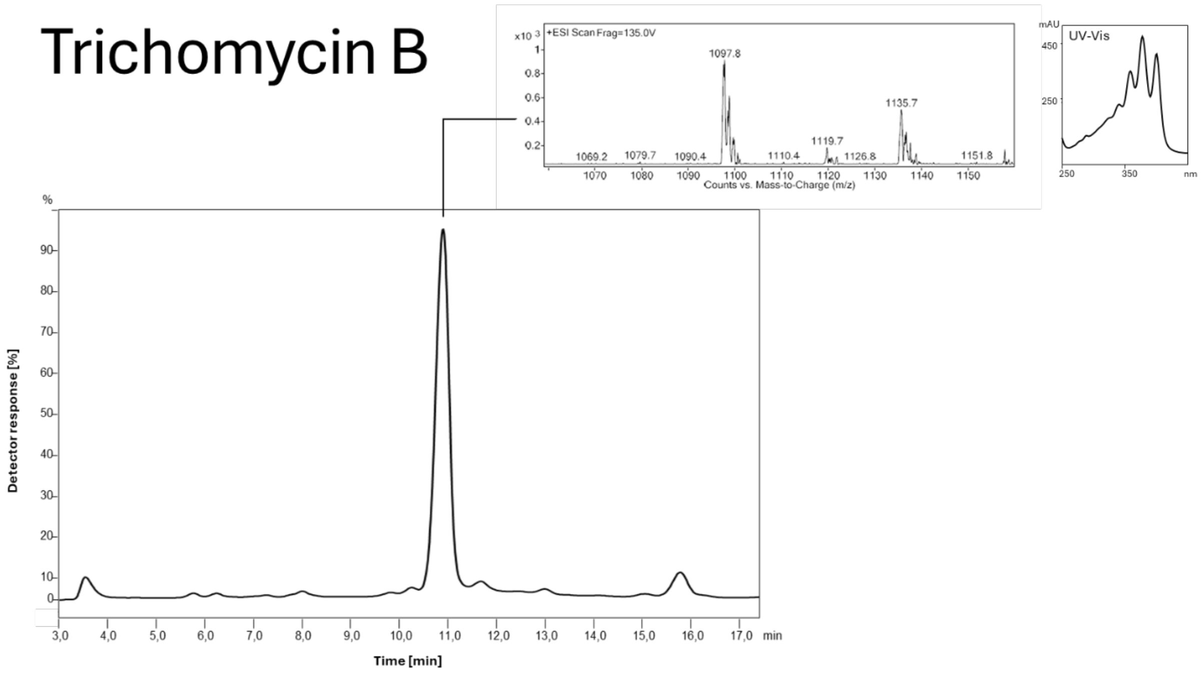

Figure S3 The HPLC-DAD-ESIMS chromatogram of isolated Trichomycin B sample. Chromatographic conditions: column Phenomenex Luna C18(2) 100 Å (150 mm × 4.6 mm, 5 µm). Mobile phase composition: 36% acetonitrile/64% ammonium acetate buffer (5.5 mmol, pH = 4.7), v/v; at a flow rate of 1 mL/min; inj. volume = 20 µL. Detection at 407 nm, room temperature.

Table *S1*. 1H and 13C NMR data for trichomycin A and B.

	Trichomycin A					Trichomycin B				
position	δ_C, type		δ_H	$J_{H,H}$ (Hz)	ROE contacts	δ_C, type		δ_H	$J_{H,H}$ (Hz)	ROE contacts
1	166.80	C	—	—	—	166.71	C	—	—	—
2	43.05	CH_2	2a: 2.454	10.5 (2b)	2b, 4a	42.98	CH_2	2a: 2.448	10.5 (2b)	2b, 4a
			2b: 2.510	10.5 (2a)	2a, 4b			2b: 2.520	10.5 (2a)	2a, 4b
3	201.98	C	—	—	—	202.04	C	—	—	—
4	51.81	CH_2	4a: 2.556	15.0 (4b), 2.3 (5)	4b, 5, 6ab*	20.23	CH_2	4a: 1.512	5a (8.0), 4b (13.5)	4b, 7
			4b: 2.824	15.0 (4a), 8.4 (5)	4a, 5, 6ab*			4b: 1.802	5b (4.0), 4a (13.5)	2b, 4a, 7
5	67.86	CH	4.096	2.3 (4a), 8.4 (4b), 10.8 (6b)	4a, 4b, 6ab*, 7a, 7b	37.77	CH_2	5a: 1.272	4a (8.0), 5b (11.5), 6a (10.0)	5b, 7
								5b: 1.389	4b (4.0), 5a (11.5), 6b (3.0)	5a, 7
6	38.51	CH_2	6ab*: 1.354	10.8 (5)	4a, 4b, 5, 7b	43.84	CH_2	6a: 1.378	5a (10.0), 6b (12.0), 7 (11.2)	6b, 7, 11
								6b: 1.617	5b (3.0), 6a (12.0), 7 (2.1)	6a
7	23.13	CH_2	7a: 1.341	13.5 (7b)	5, 7b, 9	72.22	CH	3.787	6a (11.2), 6b (2.1), 8a (10.3), 8b (3.5)	4a, 4b, 5a, 5b, 6a, 8a, 8b, 9, 28, 29
			7b: 2.047	13.5 (7a), 10.3 (8a), 2.5 (8b)	5, 6ab*, 7a, 8a, 8b, 9					
8	38.65	CH_2	8a: 1.272	10.3 (7a), 12.1 (8b), 2.5 (9)	8b, 7b, 9, 10b	43.94	CH_2	8a: 1.295	10.3 (7), 13.0 (8b), 2.5 (9)	7, 8b, 9

			8b: 1.385	2.5 (7b), 12.1 (8a), 11.5 (9)	7b, 8a, 9			8b: 1.495	3.5 (7), 13.0 (8a), 11.5 (9)	7, 8a
9	72.54	CH	3.777	2.5 (8a), 11.5 (8b), 2.5 (10a), 10.5 (10b)	7a, 7b, 8a, 10a, 10b, 11, 26, 28	72.94	CH	4.026	2.5 (8a), 11.5 (8b), 3.0 (10a), 11.0 (10b)	7, 8a, 10a, 11, 28
10	44.06	CH_2	10a: 1.353	2.5 (9), 15.0 (10a), 2.5 (11)	9, 10b, 11, 12a	44.01	CH_2	10a: 1.368	3.0 (9), 14.0 (10b), 2.5 (11)	7, 9, 10b, 11, 12a, 13
			10b: 1.520	10.5 (9), 15.0 (10a), 10.5 (11)	8a, 9, 10a, 11			10b: 1.617	11.0 (9), 14.0 (10a), 11.5 (11)	10a
11	73.15	CH	4.143	2.5 (10a), 10.5 (10b), 2.5 (12a), 12.6 (12b)	9, 10a, 10b, 12a, 13, 24, 26	72.88	CH	4.148	2.5 (10a), 11.5 (10b), 2.7 (12a), 12.3 (12b)	6a, 7, 9, 10a, 13, 24, 26
12	44.02	CH_2	12a: 1.353	2.5 (11), 14.7 (12b), 2.5 (13)	10a, 11, 12b,13, 14a	46.48	CH_2	12a: 1.614	2.7 (11), 14.2 (12b), 2.5 (13)	10a, 12b
			12b: 1.595	12.6 (11), 14.7 (12a), 11.8 (13)	12a, 13, 14b			12b: 1.798	12.3 (11), 14.2 (12a), 11.3 (13)	7, 12a, 14b
13	69.14	CH	4.592	2.5 (12a), 11.8 (12b), 2.4 (14a), 10.6 (14b)	11, 12a, 12b, 14a, 22, 23, 24	68.92	CH	4.587	2.5 (12a), 11.3 (12b), 2.6 (14a), 10.5 (14b)	10a, 11, 14a, 14b, 16a, 22, 24
14	46.78	CH_2	14a: 1.683	2.4 (13), 15.0 (14b)	12a, 13, 14b, 16a	46.74	CH_2	14a: 1.683	2.6 (13), 14.5 (14b)	13, 14b, 16a
			14b: 1.840	10.6 (13), 15.0 (14a)	12b, 14a, 16b			14b: 1.841	10.5 (13), 14.5 (14a)	12b, 13, 14a, 16b

15	97.89	C	—	—	—	98.01	C	—	—	—
16	44.97	CH_2	16a: 1.606	10.1 (16b), 10.5 (17)	14a, 16b, 18	44.91	CH_2	16a: 1.602	10.5 (16b), 10.3 (17)	12b, 13, 16b, 18
			16b: 2.381	10.1 (16a), 3.0 (17)	14b, 16a, 17			16b: 2.373	10.5 (16a), 3.3 (17)	14b, 16a, 17, 18, 19
17	66.42	CH	4.841	10.5 (16a), 3.0 (16b), ** (17)	16b, 18	66.37	CH	4.824	10.3 (16a), 3.3 (16b), ** (17)	16b, 18
18	58.36	CH	2.700	** (17), 10.1 (19)	16a, 17, 19, 20a, 20b	58.21	CH	2.691	** (17), 10.2 (19)	16a, 16b, 17, 19, 20a
19	66.31	CH	4.843	10.1 (18), 8.0 (20a)	2’, 18, 20b, 22	66.25	CH	4.825	10.2 (18), 9.0 (20a)	2’, 16b, 18, 22
20	37.48	CH_2	20a: 1.965	8.0 (19), 10.0 (20b)	18, 20b, 21	37.42	CH_2	20a: 1.971	8.2 (19), 11.0 (20b)	18, 20b, 21
			20b: 2.438	10.0 (20a), 4.6 (21)	1’, 18, 19, 20a, 21			20b: 2.429	11.0 (20a), 4.5 (21)	1’, 20a, 21
21	76.15	CH	4.794	4.6 (20b), 7.5 (22)	1’, 20a, 20b, 22, 23	76.05	CH	4.795	4.7 (20b), 7.5 (22)	1’, 20a, 20b, 22, 23
22	136.20	CH	6.185	7.5 (21), 15.0 (23)	13, 19, 21, 24	136.02	CH	6.147	7.5 (21), 15.1 (23)	13, 19, 21, 24
23	130.55	CH	6.275	15.0 (22), 10.5 (24)	13, 21, 25	133.27	CH	6.299	15.1 (22), 10.5 (24)	21, 25
24	133.63	CH	6.478	10.5 (23), ** (25)	11, 13, 22, 26	133.33	CH	6.462	10.5 (23), ** (25)	11, 13, 22, 26
25	133.10	CH	6.390	** (24), 10.0 (26)	23, 27	133.16	CH	6.415	** (24), 10.0 (26)	23, 27
26	134.92	CH	6.505	10.0 (25), 15.2 (27)	9, 11, 24, 28	134.85	CH	6.482	10.0 (25), 15.3 (27)	9, 11, 24, 28
27	128.32	CH	6.859	15.2 (26), 10.2 (28)	25, 30	128.26	CH	6.883	15.3 (26), 10.2 (28)	25, 30
28	133.40	CH	6.291	10.2 (27), 10.3 (29)	9, 26, 29	130.44	CH	6.272	10.2 (27), 10.4 (29)	7, 9, 26, 29
29	124.71	CH	6.587	10.3 (28), ** (30)	28, 32	124.69	CH	6.626	10.4 (28), ** (30)	7, 28, 32
30	124.63	CH	6.541	** (29), 10.2 (31)	27, 31, 32	124.61	CH	6.558	** (29), 10.2 (31)	27, 31, 32
31	130.32	CH	6.064	10.2 (30), 9.7 (32)	30, 33	130.36	CH	6.075	10.2 (30), 9.7 (32)	30, 33

32	127.06	CH	6.803	9.7 (31), 13.3 (33)	29, 30, 34	126.99	CH	6.748	9.7 (31), 13.5 (33)	29, 30, 34
33	134.42	CH	6.148	13.3 (32), ** (34)	31, 35	134.62	CH	6.141	13.5 (32), ** (34)	31, 35
34	132.03	CH	6.135	** (33), 15.1 (35)	32, 36	131.58	CH	6.062	** (33), 15.0 (35)	32, 36
35	137.95	CH	5.420	15.1 (34), 10.5 (36)	33, 36, Me36, 37	138.39	CH	5.441	15.0 (34), 10.5 (36)	Me36, 33, 37
36	40.02	CH	2.341	10.5 (35), 10.5 (37), 6.4 (Me36)	34, 35, Me36, 37, 38, Me38	40.29	CH	2.357	10.5 (35), 10.2 (37), 6.5 (Me36)	Me36, Me38, 34, 38
37	79.55	CH	4.857	10.5 (36), 2.3 (38)	35, 36, Me36, 38, 39b, 39a, 40ab*	79.52	CH	4.853	10.2 (36), 2.5 (38)	Me36, 35, 38, 40
38	33.46	CH	1.718	2.3 (37), 6.5 (Me38)	36, Me36, 37, Me38, 39a, 40ab*, 41	33.46	CH	1.732	2.5 (37), 6.5 (Me38)	Me36, Me38, 36, 37, 41
39	30.62	CH_2	39ab*: 1.503	** (38), ** (40ab*)	38, 37, 40ab*, 41	30.59	CH_2	39ab*: 1.485	** (38), ** (40ab*)	37, 40ab*, 41
40	35.24	CH_2	40ab*: 1.648	10.5 (41)	37, 38, Me38, 39b, 41, 42a, 42b	35.19	CH_2	40ab*: 1.646	10.5 (41)	37, 38, Me38, 39b, 41, 42a, 42b
41	68.15	CH	4.353	10.5 (40ab*), 3.2 (42a), 8.2 (42b)	38, 39a, 39b, 40ab*, 42a, 42b, 45/45’	68.10	CH	4.347	10.5 (40ab*), 3.3 (42a), 8.1 (42b)	38, 39b, 40ab*, 42a, 42b, 45/45’
42	45.71	CH_2	42a: 2.994	3.2 (41), 15.4 (42b)	39b, 40ab*, 41, 42b, 45/45’	45.66	CH_2	42a: 2.988	3.3 (41), 15.2 (42b)	40ab*, 41, 42b, 45/45’
			42b: 3.179	8.2 (41), 15.4 (42a)	39b, 40ab*, 41, 42a, 45/45’			42b: 3.181	8.1 (41), 15.2 (42a)	40ab*, 41, 42a, 45/45’
43	197.50	C	—	—	—	197.49	C	—	—	—
Me36	16.20	CH_3	0.778	6.4 (36)	35, 36, 37, 38	16.34	CH_3	0.7929	6.5 (36)	35, 36, 37, 38
Me38	12.38	CH_3	0.798	6.5 (38)	36, 38	12.30	CH_3	0.8310	6.5 (38)	36, 38, 40
**C**OOH	176.02	C	—	—	—		C	—	—	—
45/45’	131.06	CH	7.902	7.5 (46/46’)	41, 42a, 42b, 46/46’	131.1	CH	7.901	7.6 (46/46’)	41, 42a, 42b, 46/46’

46/46’	113.05	CH	6.760	7.5 (45/45’)	45/45’	113.1	CH	6.771	7.6 (45/45’)	45/45’
**C***CO	154.17	C	—	—	—	154.2	C	—	—	—
**C***NH	126.18	C	—	—	—	126.2	C	—	—	—
1'	97.16	CH	4.932	2.8 (2’)	2’, 3’, 5’, 20b, 21	97.24	CH	4.929	2.6 (2’)	2’, 3’, 5’, 20b, 21
2'	68.47	CH	4.578	2.8 (1’), 6.4 (3’)	1’, 3’, 4’, 19	68.45	CH	4.567	2.6 (1’), 6,7 (3’)	1’, 3’, 19
3'	56.71	CH	3.534	6.4 (2’), 8.5 (4’)	1’, 2’, 6’	56.70	CH	3.531	6.7 (2’), 8.4 (4’)	1’, 2’, 4’, 6’
4'	59.45	CH	3.961	8.5 (3’), 9.3 (5’)	2’, 3’, 5’, 6’	69.49	CH	3.955	8.4 (3’), 9.3 (5’)	3’, 5’, 6’
5'	73.74	CH	3.528	9.3 (4’), 5.6 (6’)	1’, 4’, 6’	73.75	CH	3.521	9.3 (5’), 5.7 (6’)	1’, 4’, 6’
6'	17.58	CH_3	1.319	5.6 (5’)	4’, 5’	17.63	CH_3	1.329	5.7 (5’)	3’, 4’, 5’

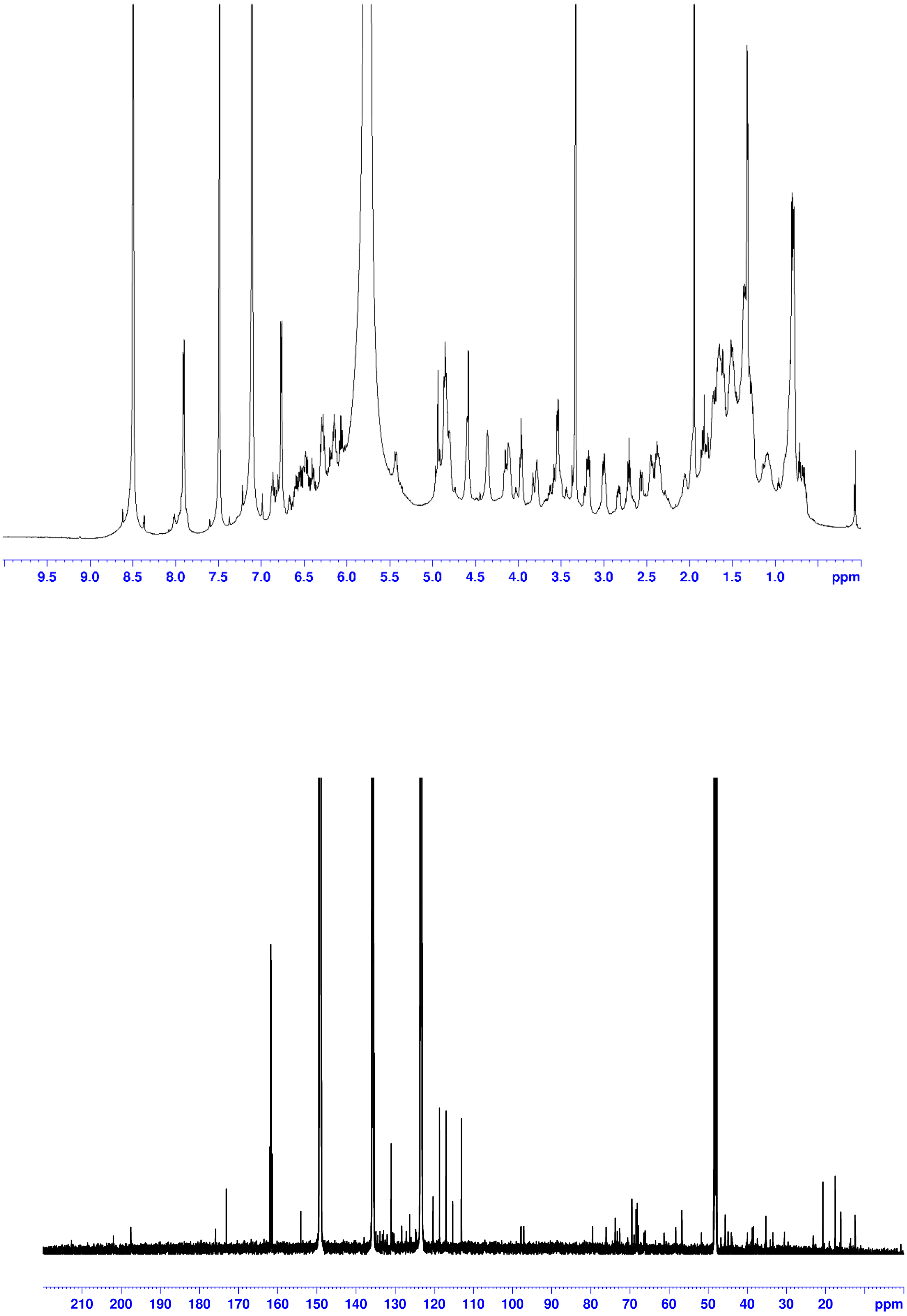

Figure *S4*. ^{1}H NMR (top) and ^{13}C NMR (bottom) spectra of trichomycin A.

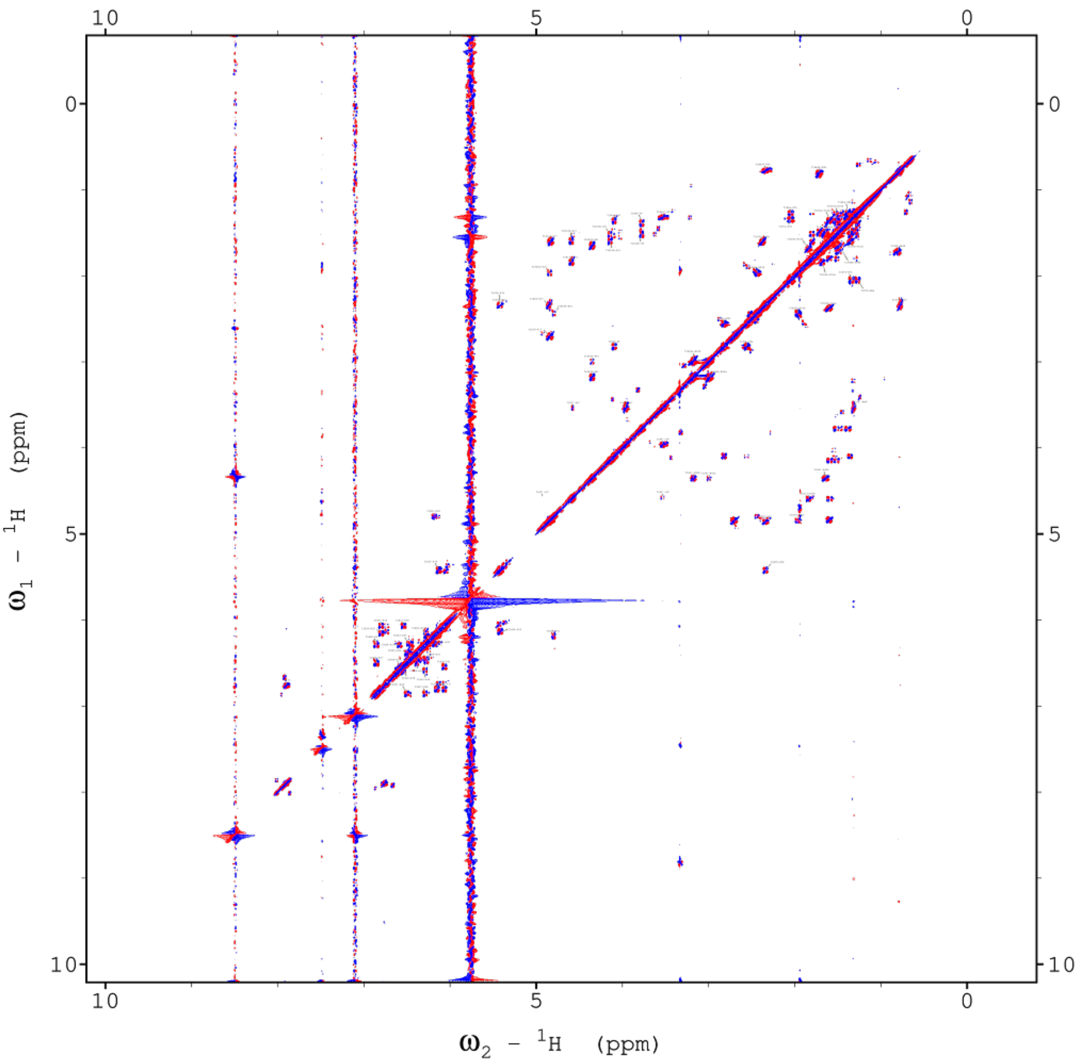

Figure *S5*. DQF-COSY spectrum of trichomycin A.

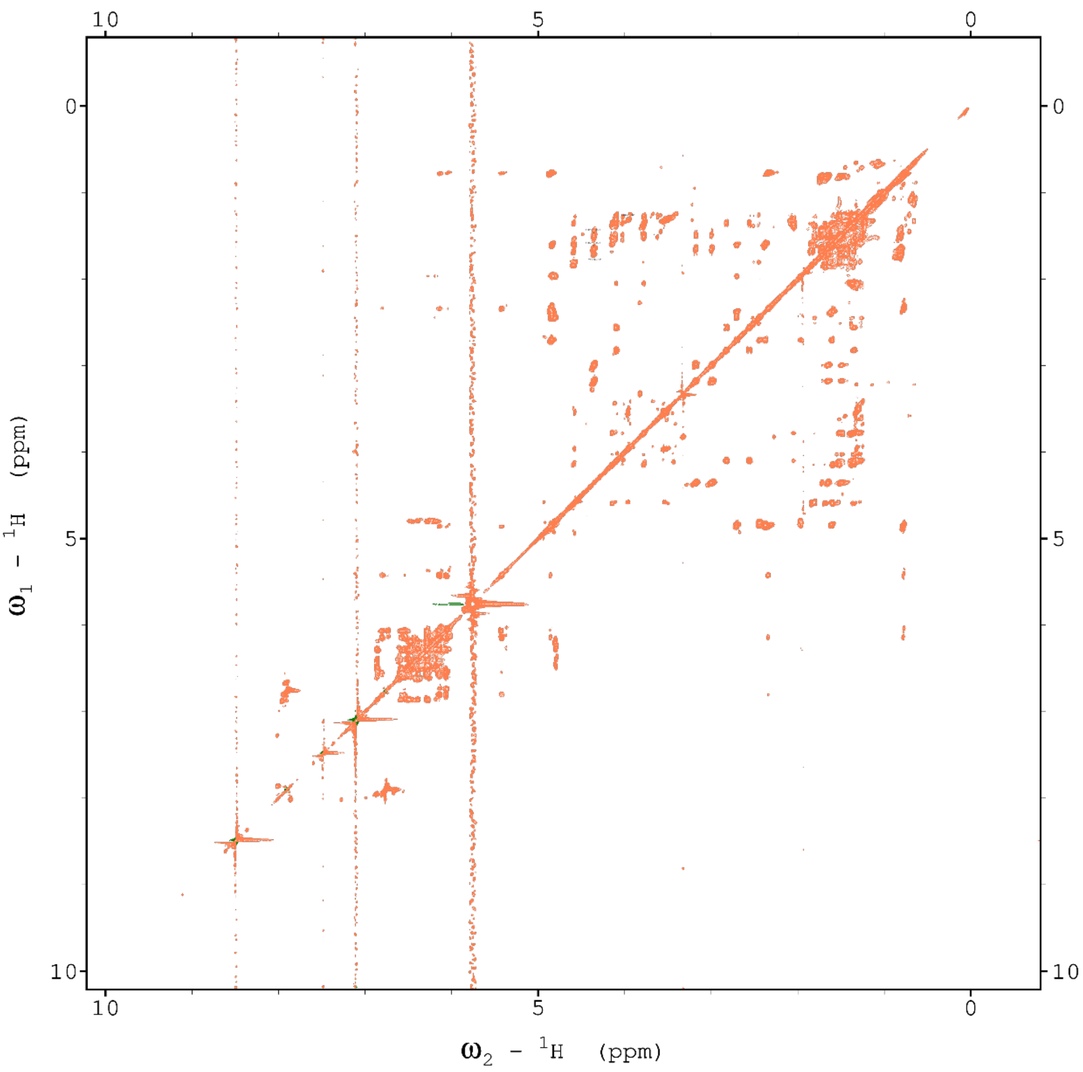

Figure *S6*. TOCSY spectrum of trichomycin A. Spin-lock = 60 ms.

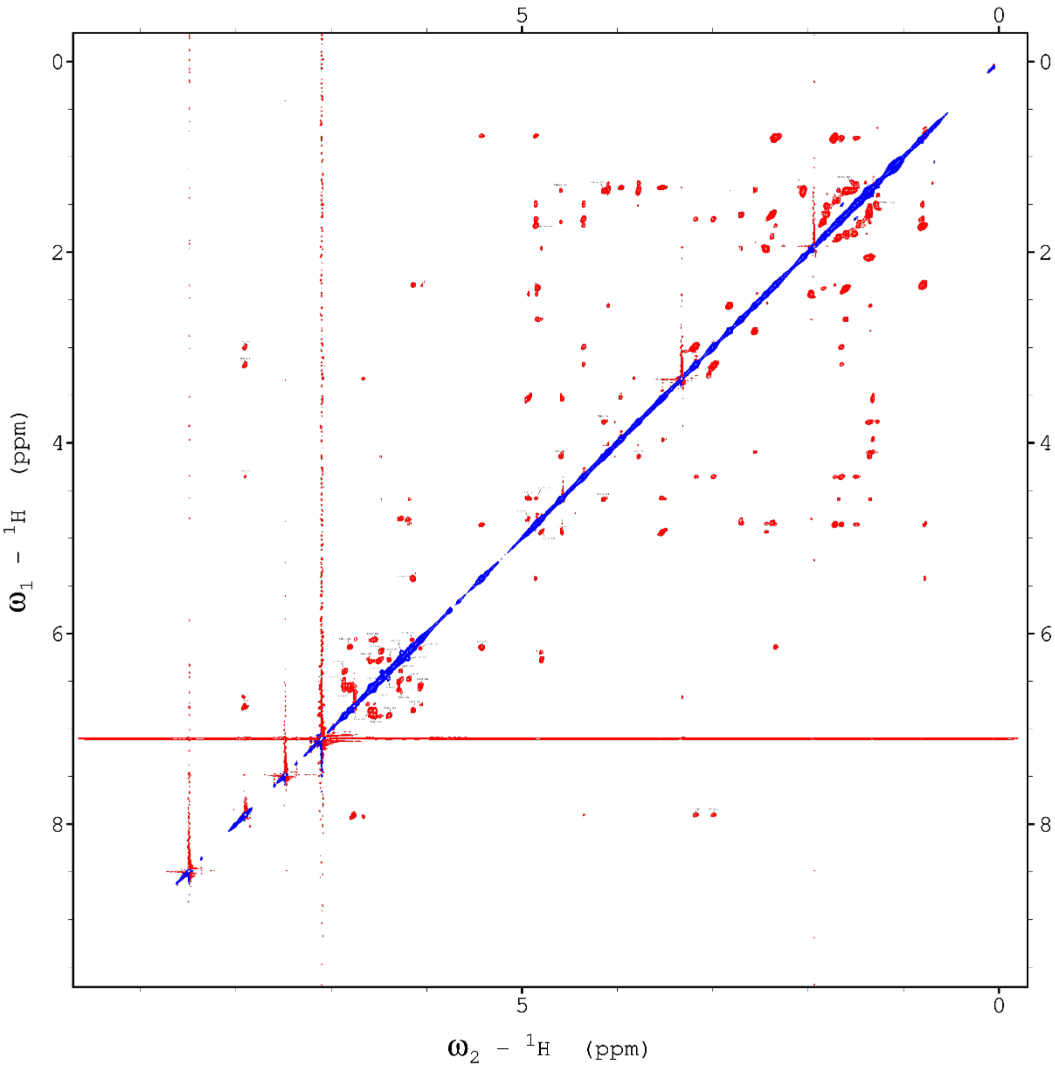

Figure *S7*. ROESY spectrum of trichomycin A. Mix time = 350 ms.

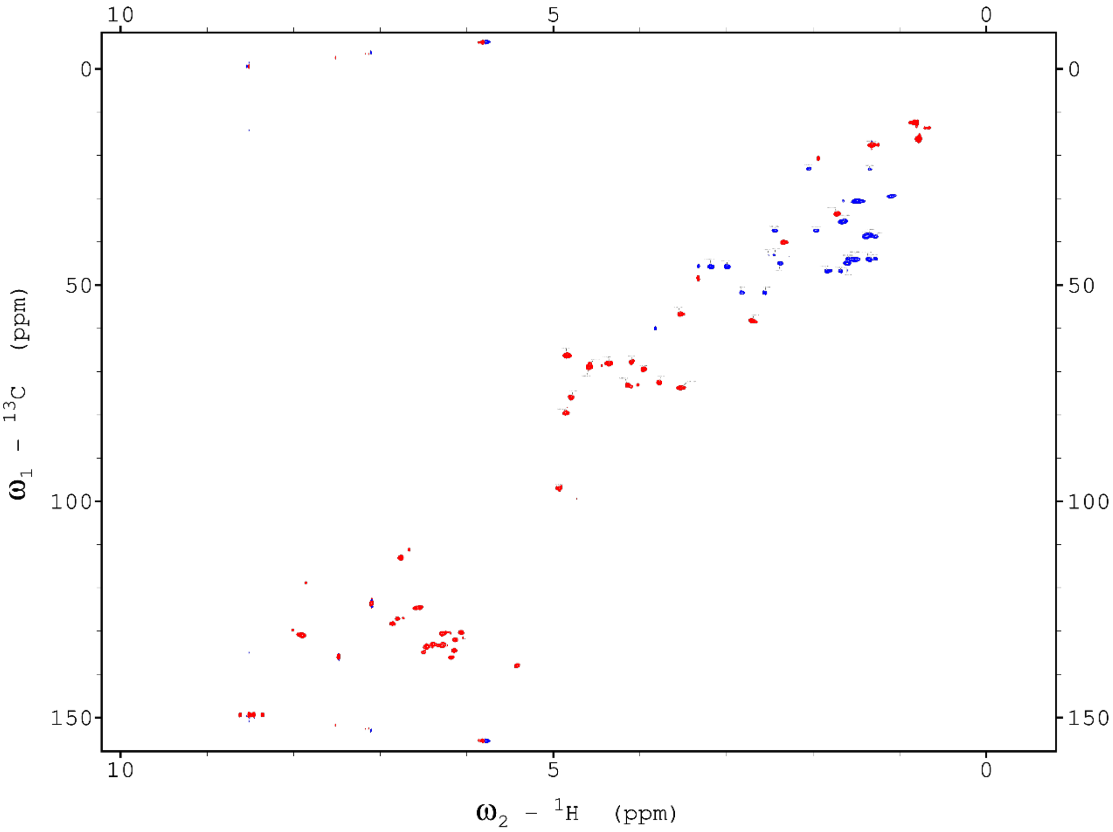

Figure *S8.* Edited-HSQC spectrum of trichomycin A.

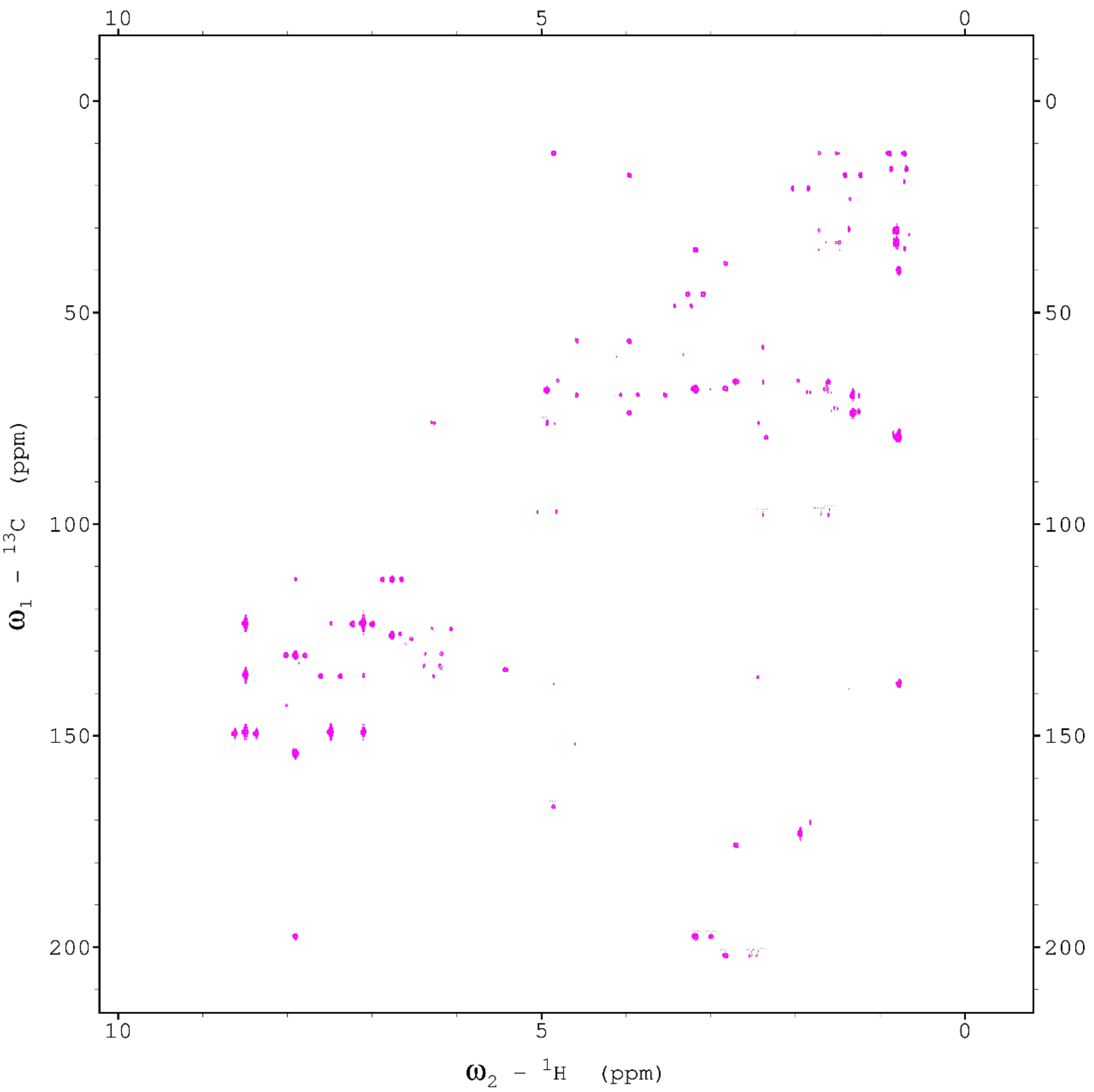

Figure *S9*. HMBC spectrum of trichomycin A.

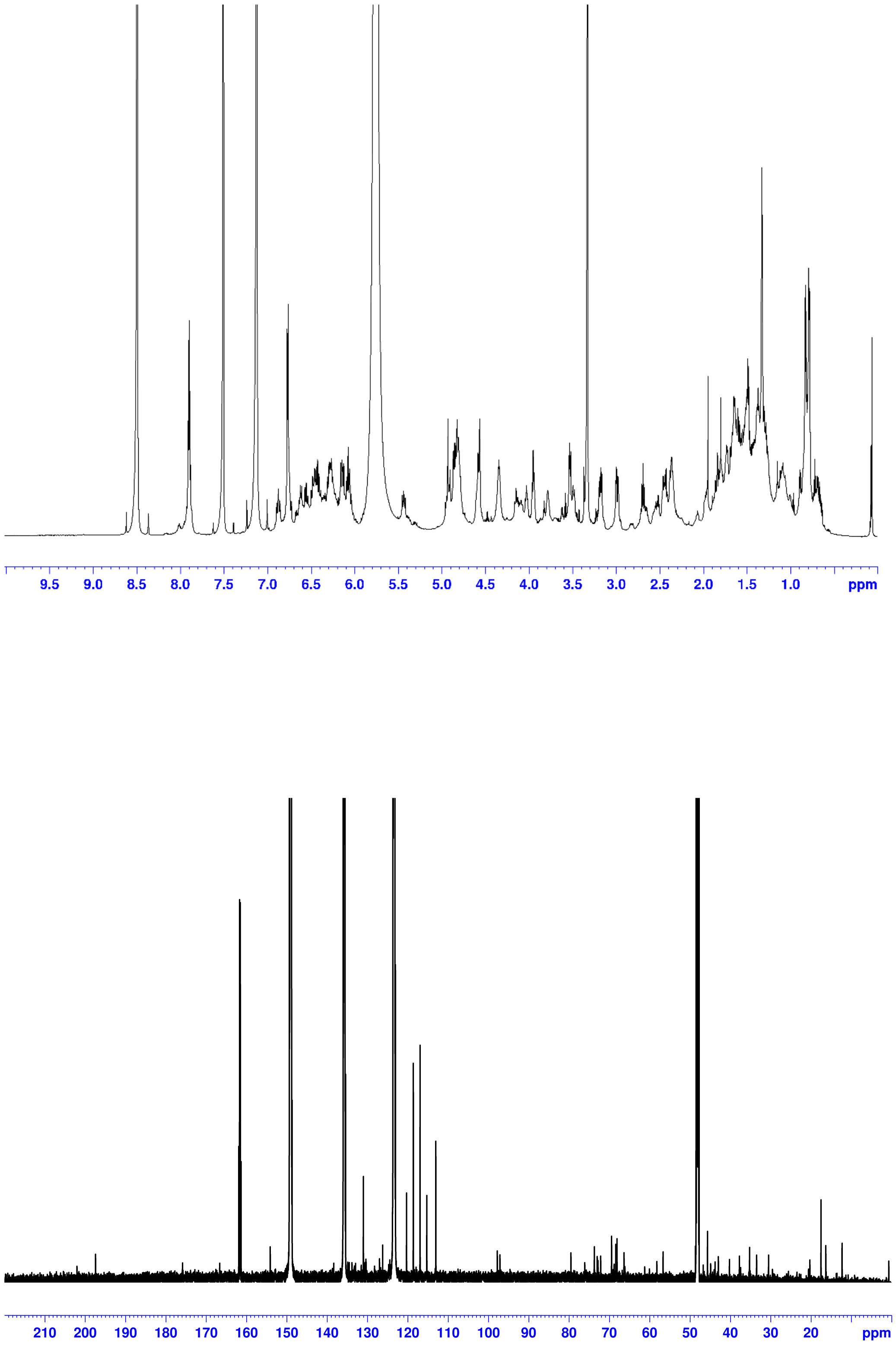

Figure *S10.* ^{1}H NMR (top) and ^{13}C NMR (bottom) spectra of trichomycin B.

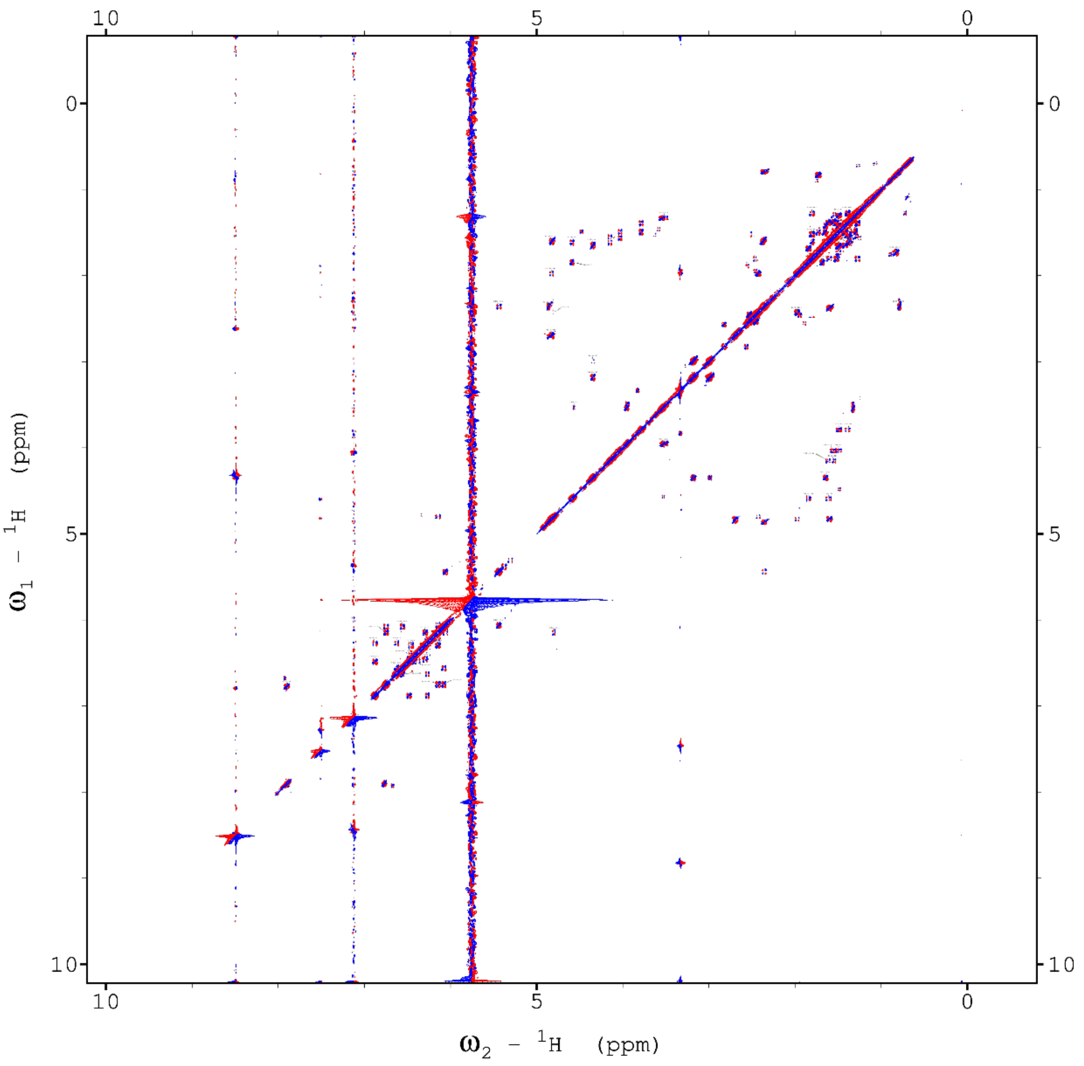

Figure *S11*. DQF-COSY spectrum of trichomycin B.

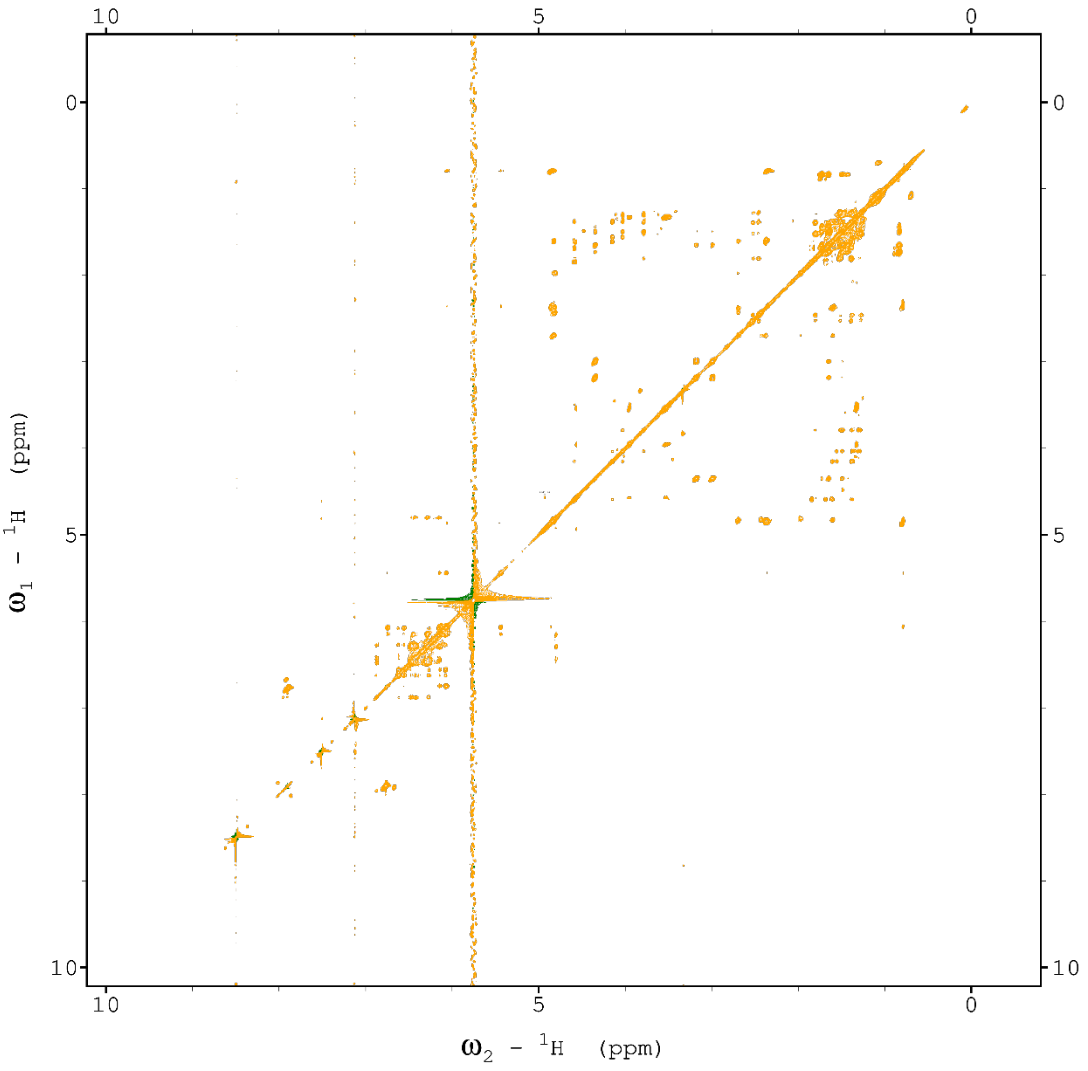

Figure *S12*. TOCSY spectrum of trichomycin B. Spin-lock = 60 ms.

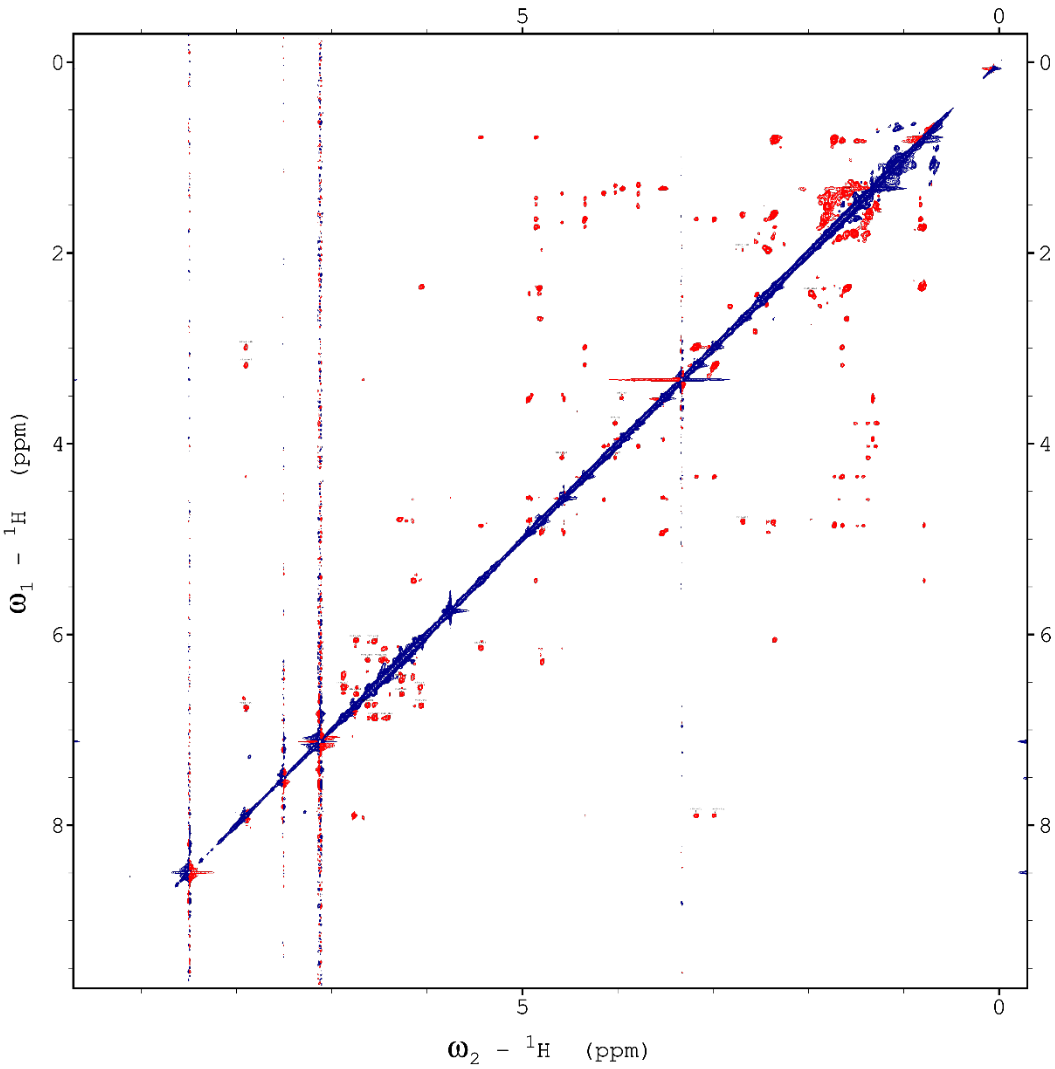

Figure *S13.* ROESY spectrum of trichomycin B. Mix time = 350 ms.

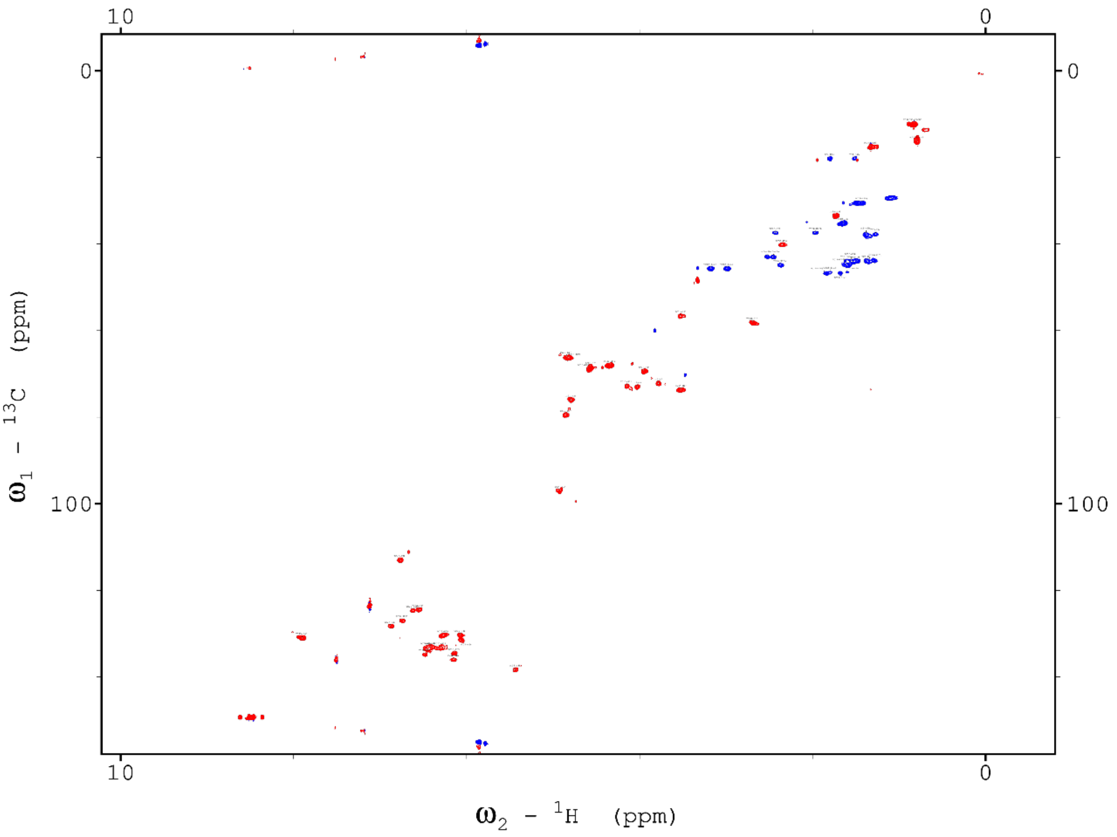

Figure *S14*. Edited-HSQC spectrum of trichomycin B.

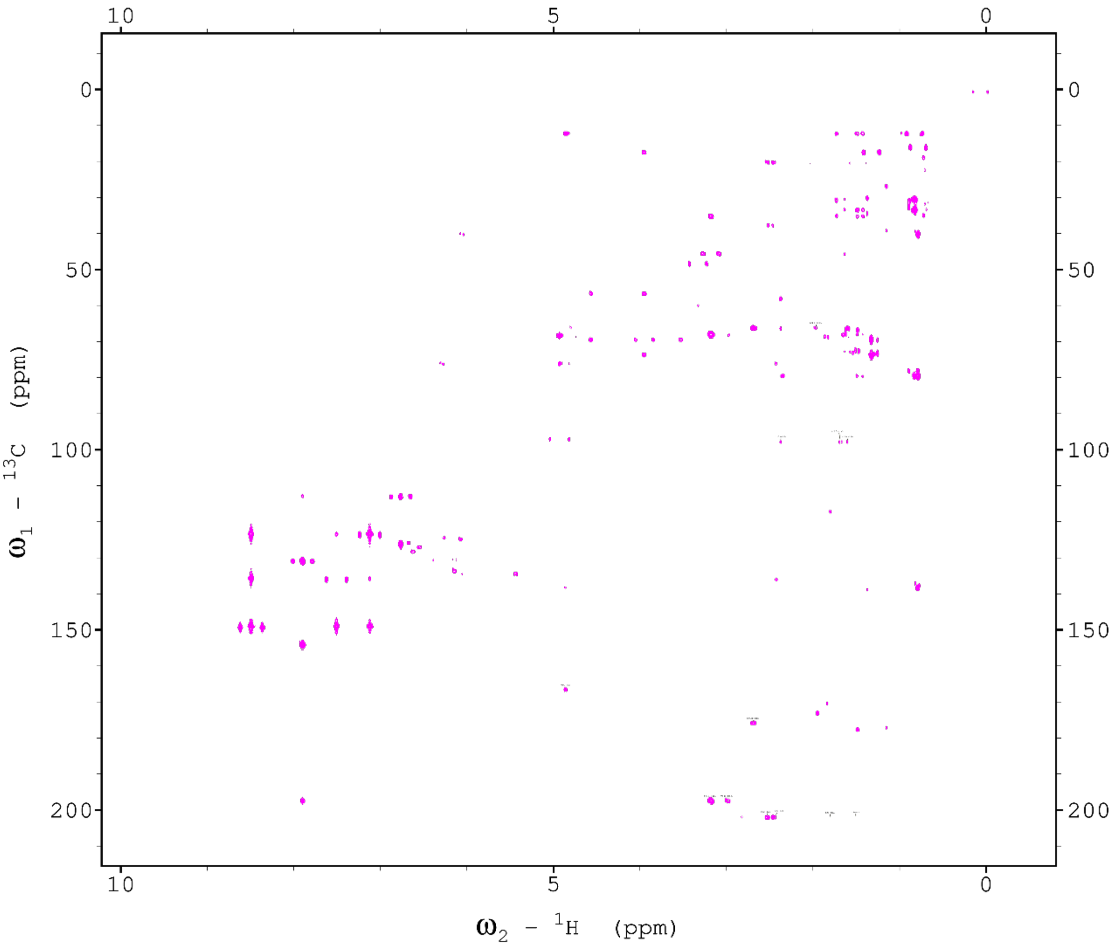

Figure *S15*. HMBC spectrum of trichomycin B.

Table S2 Convergence data for QM calculations for all studied molecules.

	Item	Value	Threshold	Converged?	Value	Threshold	Converged?
Trychomycin A		41R			41S		
	Maximum Force	0.000068	0.000450	YES	0.000019	0.000450	YES
	RMS Force	0.000007	0.000300	YES	0.000002	0.000300	YES
	Maximum Displacement	0.001766	0.001800	YES	0.001201	0.001800	YES
	RMS Displacement	0.000329	0.001200	YES	0.000274	0.001200	YES
	Predicted change in Energy	-1.741831D-08			-5.106106D-09		
Trychomycin B		41R			41S		
	Maximum Force	0.000007	0.000450	YES	0.000015	0.000450	YES
	RMS Force	0.000001	0.000300	YES	0.000001	0.000300	YES
	Maximum Displacement	0.001051	0.001800	YES	0.001325	0.001800	YES
	RMS Displacement	0.000149	0.001200	YES	0.000189	0.001200	YES
	Predicted change in Energy	-1.883605D-09			-2.459863D-09		

Table S3 The XYZ coordinates for both enantiomers of Trichomycin A and B.

Trichomycin A 41R			
C	-7.77660	2.04479	-0.28426
C	-7.57658	1.55281	-1.68112
C	-7.63005	0.04759	-1.75909
H	-6.63168	1.93210	-2.07678
H	-8.37165	1.94579	-2.32615
C	-6.56913	-0.80107	-1.07290
C	-5.14769	-0.26804	-1.19846
H	-6.62443	-1.79661	-1.53170
H	-6.83444	-0.91237	-0.01813
C	-4.14372	-1.22471	-0.53481
H	-2.60801	0.22112	-0.07885
C	-2.70521	-0.71090	-0.64793
H	-4.20394	-2.20680	-1.02038
H	-4.40155	-1.36323	0.52223
C	-1.68859	-1.73638	-0.12914
C	-0.23801	-1.26310	-0.30041
H	-1.82809	-2.67815	-0.67406
H	-1.89014	-1.93236	0.93091
C	0.75125	-2.23086	0.36883
H	-0.11601	-0.26760	0.13892
O	0.10106	-1.18192	-1.68915
C	2.21188	-1.78072	0.19355
H	0.63640	-3.22198	-0.08899
H	0.51294	-2.31647	1.43508
C	3.17089	-2.66367	1.00695
H	2.31811	-0.73982	0.51824
O	2.59272	-1.84522	-1.18595
C	4.64278	-2.27813	0.78748
H	3.03572	-3.70749	0.69529
H	2.91577	-2.58836	2.07002
C	5.56915	-3.01891	1.76688
H	4.76010	-1.19655	0.91270
O	5.02955	-2.61168	-0.55267
C	7.06107	-2.70171	1.53822
H	5.41837	-4.09951	1.64721
H	5.28212	-2.74172	2.78859
C	7.92143	-3.35275	2.61759
O	7.23140	-1.29074	1.61324
O	7.46103	-3.19080	0.26161
C	9.38715	-2.95412	2.45005
H	7.84000	-4.44634	2.57967
H	7.58277	-3.04579	3.61617
C	9.55775	-1.43054	2.38613
H	9.77440	-3.43988	1.54552
O	10.11230	-3.48982	3.56285
C	8.57262	-0.84923	1.33773
C	11.01640	-1.08410	2.04522
H	9.33681	-1.01144	3.37703
C	8.48788	0.69365	1.29452
C	8.05727	1.32190	-0.06209
H	9.44309	1.14380	1.55501
H	7.78440	1.03399	2.06708
C	6.64868	0.97070	-0.44911
C	5.62777	1.83716	-0.37293
C	4.27411	1.50742	-0.74993
C	3.25717	2.35998	-0.55783
C	1.88857	2.05695	-0.90726
C	0.87306	2.88302	-0.61201
C	-1.61678	4.63852	-0.05873
C	-2.69420	5.28449	0.41269
C	-4.02012	4.73667	0.58638
C	-5.10747	5.49265	0.80331
C	-6.40738	4.90180	1.02402
C	-7.55895	5.58575	0.95969
C	-8.97261	5.04085	1.14051
C	-9.13214	3.48583	1.08449

H	-9.53688	5.46182	0.29763
C	-9.52900	5.63081	2.44228
O	-8.71106	3.03280	-0.23246
C	-10.58960	2.96157	1.31886
H	-8.49429	3.06389	1.86867
O	-7.17397	1.58831	0.67842
H	-0.67944	5.19064	-0.06943
H	-6.40604	3.84330	1.26120
H	-7.51675	6.65175	0.73348
H	-4.11992	3.65386	0.55874
H	-5.03650	6.57759	0.80951
C	-1.59066	3.30885	-0.62980
H	-2.55919	6.31580	0.73717
H	1.70713	1.11120	-1.41300
H	4.10320	0.52747	-1.19183
H	3.44609	3.32947	-0.10122
H	6.48581	-0.04598	-0.80124
H	5.80926	2.84448	-0.00425
H	8.83434	-1.21803	0.34255
H	-0.54846	-0.61085	-2.13437
H	1.79454	-1.58701	-1.70149
H	4.28292	-2.31164	-1.12256
H	6.68489	-3.04421	-0.32246
H	11.05430	-3.38372	3.31920
O	11.35320	0.13116	2.14574
O	11.76510	-1.97495	1.54102
H	-9.04514	5.18328	3.31763
H	-10.60690	5.46932	2.53259
H	-9.36940	6.71456	2.48876
C	-10.72400	1.42839	1.12733
C	-11.60150	3.63140	0.37308
H	-10.88560	3.19432	2.34912
C	-9.95743	0.57960	2.14792
H	-10.42460	1.16471	0.10583
H	-11.78430	1.15247	1.20335
C	-9.50280	-0.76639	1.56984
C	-10.64880	-1.69641	1.17195
O	-8.68827	-1.40917	2.55173
H	-8.87216	-0.56841	0.70167
C	-10.12080	-2.89623	0.39824
H	-11.35510	-1.15939	0.53219
H	-11.15770	-2.07025	2.06701
C	-11.09790	-3.78334	-0.30174
C	-10.61020	-4.81244	-1.11489
C	-11.49510	-5.67409	-1.77255
C	-12.87550	-5.50970	-1.65025
C	-13.35700	-4.50141	-0.81359
C	-12.48000	-3.63595	-0.14919
H	-9.53865	-4.95731	-1.23755
H	-11.09120	-6.47755	-2.38312
N	-13.74790	-6.40589	-2.24298
H	-14.42760	-4.38123	-0.66691
H	-12.90740	-2.87030	0.49043
C	-0.50595	2.56954	-0.91062
O	-8.59478	-0.50155	-2.30519
O	-8.91973	-3.17002	0.36731
H	-8.28112	-2.17546	2.09548
H	-11.33540	3.47031	-0.67688
H	-11.66890	4.70874	0.54629
H	-12.60690	3.22496	0.52965
C	10.24010	1.36031	-1.06826
C	11.03120	0.06172	-0.93797
C	12.47900	0.41667	-0.67717
O	10.96580	-0.77293	-2.11272
H	10.62720	-0.55436	-0.16204
C	13.01210	1.17177	-1.91327
H	12.61780	1.03801	0.21476
N	13.27010	-0.84213	-0.41125
C	12.10220	2.41005	-2.15033
H	12.98680	0.51620	-2.79248

O	14.38510	1.48714	-1.66395
O	10.70870	2.05033	-2.23548
C	12.44900	3.12501	-3.45218
H	12.22780	3.12837	-1.33006
H	10.01100	-0.79857	-2.34114
H	14.72380	1.96340	-2.44577
H	14.23870	-0.60218	-0.34069
H	-14.73810	-6.20072	-2.25974
H	12.33530	2.45138	-4.30898
H	13.47000	3.51526	-3.44372
H	11.76100	3.96129	-3.62141
H	10.38610	2.02529	-0.20879
O	8.87150	0.98092	-1.19776
H	8.12160	2.41288	0.05370
H	13.07940	-1.39845	-0.96774
H	-9.07282	1.10386	2.52237
H	-10.58090	0.41377	3.03558
H	-0.67869	1.61105	-1.39311
H	-2.55512	2.88191	-0.90132
H	-4.86444	-0.99938	-2.99701
H	-5.05963	0.72053	-0.73635
O	-4.82455	-0.11857	-2.58076
H	1.07974	3.81525	-0.09403
H	-2.49357	-0.47364	-1.69447
H	-13.39910	-7.04916	-2.94149
H	13.04580	-1.08308	0.17633
**Trichomycin A 41S**			
C	7.65940	2.00170	0.20610
C	7.49440	1.67440	1.66450
C	7.35060	0.18930	1.98760
H	6.56180	2.16180	1.99850
H	8.32190	2.08140	2.25300
C	6.33590	-0.64340	1.23280
C	4.93770	-0.01870	1.21000
H	6.31160	-1.62300	1.73420
H	6.69420	-0.78990	0.20480
C	3.89830	-1.02690	0.72370
H	2.36590	0.39640	0.16440
C	2.46570	-0.51260	0.78150
H	3.97760	-1.93780	1.34520
H	4.16340	-1.33790	-0.30000
C	1.44710	-1.55530	0.32820
C	0.01760	-1.18870	0.71490
H	1.67570	-2.52540	0.80110
H	1.51240	-1.70500	-0.76150
C	-0.99260	-2.22460	0.21620
H	-0.23990	-0.21250	0.24920
O	0.00540	-1.06770	2.12430
C	-2.44970	-1.84500	0.47090
H	-0.78890	-3.19160	0.70570
H	-0.85010	-2.36220	-0.86670
C	-3.41770	-2.87440	-0.11370
H	-2.65070	-0.86370	-0.00910
O	-2.60880	-1.71920	1.87770
C	-4.88960	-2.48980	0.01810
H	-3.25480	-3.84340	0.38610
H	-3.18390	-3.00670	-1.18070
C	-5.79090	-3.52780	-0.65420
H	-5.05570	-1.51150	-0.47200
O	-5.16300	-2.36880	1.40980
C	-7.28190	-3.19990	-0.59060
H	-5.63710	-4.51370	-0.19180
H	-5.50730	-3.60080	-1.71330
C	-8.15550	-4.15290	-1.40710
O	-7.41040	-1.89210	-1.09160
O	-7.64190	-3.24590	0.78270
C	-9.59090	-3.65020	-1.45480

H	-8.11660	-5.16630	-0.98480
H	-7.76950	-4.19940	-2.43510
C	-9.63980	-2.21110	-1.98090
H	-10.01590	-3.62180	-0.42740
O	-10.34310	-4.53570	-2.23600
C	-8.72360	-1.35080	-1.07100
C	-11.09210	-1.69340	-2.04920
H	-9.21730	-2.17580	-2.99770
C	-8.58230	0.11600	-1.47240
C	-8.22250	1.10210	-0.33840
H	-9.49150	0.43630	-1.99540
H	-7.77050	0.17020	-2.21190
C	-6.96030	0.73640	0.37500
C	-5.85720	1.50120	0.33400
C	-4.58180	1.17930	0.92440
C	-3.50420	1.99360	0.80780
C	-2.18940	1.72490	1.31010
C	-1.13510	2.55250	1.08210
C	1.29110	4.31720	0.57670
C	2.35740	4.96730	0.04090
C	3.65950	4.41230	-0.20470
C	4.71490	5.14340	-0.63270
C	6.01740	4.59330	-0.92440
C	7.10260	5.35230	-1.14260
C	8.51810	4.93940	-1.43640
C	8.82440	3.44000	-1.31150
H	9.14670	5.46680	-0.69780
C	8.89900	5.45200	-2.83370
O	8.51260	3.02020	0.03160
C	10.30960	3.09230	-1.57150
H	8.18020	2.87900	-2.00460
O	7.05150	1.46250	-0.69190
H	0.34250	4.85820	0.60150
H	6.08240	3.50410	-0.95710
H	6.97270	6.44130	-1.11110
H	3.79450	3.33490	-0.06430
H	4.59320	6.22630	-0.74960
C	1.30910	3.00520	1.16690
H	2.21650	6.00510	-0.27500
H	-2.03170	0.79180	1.86210
H	-4.49250	0.23140	1.46560
H	-3.62790	2.93260	0.25470
H	-6.94790	-0.22260	0.90080
H	-5.90490	2.45310	-0.20870
H	-9.10230	-1.41110	-0.02980
H	-0.90680	-1.21190	2.42810
H	-3.54940	-1.84150	2.09660
H	-6.11720	-2.47150	1.55950
H	-8.59420	-3.39180	0.87620
H	-11.26440	-4.23690	-2.14700
O	-11.34700	-0.66060	-2.67100
O	-11.94570	-2.36130	-1.34830
H	8.36690	4.88320	-3.60940
H	9.97610	5.38020	-3.03090
H	8.61230	6.50570	-2.94550
C	10.64960	1.64670	-1.18290
C	11.26270	4.02450	-0.81960
H	10.47380	3.20550	-2.65690
C	9.90120	0.53570	-1.91950
H	10.48770	1.53180	-0.10040
H	11.73240	1.51750	-1.34200
C	9.93720	-0.78440	-1.15100
C	11.36010	-1.31870	-0.96560
C	11.38420	-2.67210	-0.27470
H	11.94970	-0.61210	-0.35840
H	11.86820	-1.39370	-1.94000

C	12.65780	-3.41720	-0.18010
C	12.65280	-4.67640	0.43910
C	13.81160	-5.41310	0.56900
C	15.03290	-4.91040	0.08500
C	15.04530	-3.64880	-0.53230
C	13.87600	-2.92130	-0.66170
H	11.70540	-5.05870	0.81980
H	13.78990	-6.39340	1.04750
N	16.18280	-5.66450	0.16680
H	15.98580	-3.24830	-0.91350
H	13.92140	-1.94210	-1.14000
C	0.21320	2.25220	1.46440
O	8.01130	-0.28420	2.88490
O	10.35460	-3.13320	0.20310
H	11.02850	4.02470	0.25460
H	11.22600	5.06210	-1.17460
H	12.29930	3.68050	-0.93220
C	-10.51770	1.52520	0.18260
C	-11.36240	0.26760	0.32070
C	-12.73620	0.54720	-0.23160
O	-11.50410	-0.10730	1.67170
H	-10.88390	-0.53230	-0.23810
C	-13.35000	1.67970	0.56950
H	-12.64890	0.83190	-1.29010
N	-13.52470	-0.71040	-0.21620
C	-12.37950	2.88720	0.50910
H	-13.46110	1.36940	1.62370
O	-14.59710	1.96390	-0.02520
O	-11.08150	2.54320	0.96580
C	-12.85590	4.04550	1.35930
H	-12.32920	3.19770	-0.55520
H	-10.63870	0.02200	2.09530
H	-15.11120	2.53180	0.56590
H	-14.47890	-0.54040	-0.55460
H	-12.95660	-1.48510	-0.84510
H	-12.93340	3.73990	2.41110
H	-13.83100	4.41310	1.01500
H	-12.14210	4.87440	1.29670
H	-10.48540	1.85210	-0.87690
O	-9.25250	1.22750	0.66800
H	-8.09690	2.09730	-0.80420
H	-13.57370	-1.07380	0.74500
H	8.84310	0.79280	-2.05600
H	10.32600	0.38360	-2.92430
H	0.36120	1.30110	1.98520
H	2.28490	2.58980	1.43610
H	4.54340	-0.20740	3.11690
H	4.94270	0.85260	0.53570
O	4.60620	0.52250	2.48050
H	-1.32250	3.46760	0.51120
H	2.23720	-0.21420	1.81440
H	16.19580	-6.40010	0.86480
H	17.06290	-5.16470	0.10140
O	9.32240	-0.54990	0.09830
H	9.36380	-1.54120	-1.71870
H	9.31310	-1.39970	0.57070
**Trichomycin B 41R**			
C	-7.49121	-0.68911	-0.00356
C	-6.91463	0.60301	-0.53306
C	-6.12437	0.46859	-1.82970
H	-6.26562	1.01038	0.26055
H	-7.71619	1.33637	-0.68492
C	-4.99694	-0.54494	-1.91017
C	-4.20147	-0.73250	-0.61856
H	-4.37753	-0.23704	-2.76257
H	-5.45548	-1.50864	-2.18225

C	-3.09452	-1.78618	-0.68830
C	-1.78815	-1.34352	-1.34677
H	-3.45764	-2.69039	-1.20161
H	-2.84850	-2.09258	0.34143
C	-0.64087	-2.28278	-1.00507
C	0.69429	-1.83948	-1.60264
H	-0.87074	-3.29924	-1.36236
H	-0.55376	-2.32668	0.09065
C	1.88240	-2.46705	-0.88891
H	0.77559	-0.73616	-1.48789
O	0.79384	-2.16958	-2.97711
C	3.22114	-1.90348	-1.35084
H	1.86539	-3.55891	-1.03446
H	1.77797	-2.26688	0.18930
C	4.36773	-2.35083	-0.45959
H	3.15237	-0.79738	-1.29957
O	3.55501	-2.28906	-2.67571
C	5.72634	-1.71632	-0.84020
H	4.43049	-3.44867	-0.51140
H	4.11666	-2.08562	0.57846
C	6.75680	-2.71989	-1.35960
H	6.15428	-1.24709	0.05359
O	5.56817	-0.63411	-1.76518
C	8.15231	-2.09291	-1.49131
H	6.44167	-3.12426	-2.33418
H	6.81992	-3.56066	-0.65363
C	9.25436	-3.09069	-1.83597
O	8.40695	-1.55053	-0.20253
O	8.18009	-1.09247	-2.46926
C	10.61160	-2.41211	-1.73544
H	9.10353	-3.48114	-2.85107
H	9.21610	-3.93426	-1.13111
C	10.81205	-1.80739	-0.34013
H	10.64163	-1.58202	-2.46399
O	11.60875	-3.35933	-2.03443
C	9.62963	-0.84556	-0.07098
C	12.18772	-1.11223	-0.21864
H	10.76710	-2.60736	0.41768
C	9.59159	-0.22981	1.32685
C	8.87764	1.13408	1.44091
H	10.61040	-0.17214	1.72791
H	9.04618	-0.93168	1.97469
C	7.46951	1.07996	0.93455
C	6.38264	1.04067	1.72361
C	5.03993	0.88954	1.21794
C	3.93381	0.74067	1.98921
C	2.62070	0.56729	1.44254
C	1.47594	0.36639	2.14843
C	-1.33060	-0.00558	3.46150
C	-2.56291	-0.20949	4.00217
C	-3.77130	-0.54976	3.30258
C	-4.98941	-0.59337	3.89211
C	-6.21918	-0.94132	3.22053
C	-7.42788	-0.83155	3.79774
C	-8.78704	-1.17824	3.24812
C	-8.75058	-1.61469	1.78216
H	-9.40311	-0.26198	3.30366
C	-9.42617	-2.22946	4.16469
O	-8.23991	-0.45873	1.07285
C	-10.07021	-2.02523	1.10062
H	-8.02451	-2.43478	1.65797
O	-7.27978	-1.78805	-0.46456
H	-1.52643	-0.33319	-0.96818
H	-6.12085	-1.30663	2.19246
H	-7.45668	-0.46122	4.82965
H	-3.70559	-0.79394	2.23731

H	-5.06787	-0.33021	4.95275
C	-1.01800	0.00610	2.06250
H	-2.66093	-0.10915	5.08680
H	2.54579	0.60415	0.34873
H	4.92831	0.86774	0.12700
H	4.03378	0.73976	3.07979
H	7.35443	1.00747	-0.15242
H	6.50509	1.09767	2.81102
H	9.64420	-0.05194	-0.84238
H	-0.01929	-1.85093	-3.40423
H	2.74682	-2.21535	-3.21412
H	5.05703	-0.98948	-2.51786
H	7.35177	-0.58693	-2.37187
H	12.43136	-2.84212	-2.07178
O	12.57885	-0.74417	0.90155
O	12.79852	-0.88072	-1.31848
H	-8.95970	-3.21436	4.02217
H	-10.50465	-2.32595	3.99590
H	-9.28511	-1.94778	5.21635
C	-11.18952	-1.01112	1.34800
C	-10.48628	-3.47004	1.38368
H	-9.82359	-1.98861	0.02510
C	-12.41589	-1.23312	0.46858
H	-10.80888	0.00582	1.16741
H	-11.50395	-1.04327	2.40523
C	-13.41611	-0.08184	0.55907
C	-14.68445	-0.35606	-0.25940
O	-12.82899	1.15096	0.19145
H	-13.72121	0.06055	1.60843
C	-15.66474	0.79424	-0.08020
H	-15.17458	-1.25941	0.12725
H	-14.41840	-0.54765	-1.30885
C	-15.78273	1.85825	-1.10491
C	-15.03903	1.89791	-2.29064
C	-15.18056	2.93520	-3.19611
C	-16.08420	3.97919	-2.94548
C	-16.83767	3.94444	-1.75849
C	-16.68437	2.90644	-0.86316
H	-14.33033	1.10479	-2.53053
H	-14.59443	2.94307	-4.11626
N	-16.27241	4.98635	-3.86839
H	-17.54728	4.74775	-1.55307
H	-17.26056	2.88097	0.06203
H	-15.51601	5.14725	-4.52478
H	-16.67944	5.84761	-3.51977
O	-16.31746	0.83731	0.94919
H	-12.56853	1.10034	-0.74132
H	-10.98101	-3.59243	2.35512
H	-9.62091	-4.14667	1.35574
H	-11.19052	-3.81847	0.61707
C	10.87636	2.34976	0.95382
C	11.64164	1.92876	-0.29521
C	13.11088	2.05389	0.01253
O	11.36753	2.77690	-1.38841
H	11.39127	0.89984	-0.54991
C	13.41844	3.50366	0.32317
H	13.35863	1.40715	0.86608
N	13.88899	1.50610	-1.13323
C	12.50735	3.92504	1.50438
H	13.18199	4.13663	-0.55043
O	14.79090	3.55972	0.64949
O	11.13813	3.70587	1.21621
C	12.66052	5.39226	1.84451
H	12.81190	3.30300	2.37173
H	10.40447	2.78212	-1.51289
H	15.07596	4.48401	0.67523

H	14.88940	1.68381	-1.00468
H	13.67577	0.49918	-1.19676
H	12.38465	6.01355	0.98214
H	13.69079	5.62347	2.14437
H	12.00015	5.65405	2.67881
H	11.20959	1.74158	1.81774
O	9.52276	2.18955	0.70242
H	8.87309	1.42141	2.50931
H	13.57173	1.94929	-2.00926
H	-12.10281	-1.34578	-0.58656
H	-12.92550	-2.17163	0.73722
O	-1.86735	-1.29410	-2.77045
H	-2.35347	-0.50296	-3.03934
O	-6.39215	1.18505	-2.76772
C	0.20875	0.19785	1.50129
H	1.52809	0.32621	3.24003
H	-0.51381	0.20939	4.15420
H	-1.85864	-0.10221	1.37138
H	0.25001	0.24007	0.40799
H	-4.90406	-1.04001	0.16946
H	-3.77062	0.23058	-0.28875
**Trichomycin B 41S**			
C	-7.28573	-0.19990	0.09341
C	-6.66779	0.24407	-1.21210
C	-5.86783	-0.82580	-1.94603
H	-6.01455	1.10185	-0.97793
H	-7.44635	0.62226	-1.88599
C	-4.77613	-1.57822	-1.20601
C	-3.99676	-0.74992	-0.18499
H	-4.14049	-2.01984	-1.98472
H	-5.26505	-2.41356	-0.68062
C	-2.93080	-1.52016	0.59640
C	-1.60899	-1.76425	-0.13190
H	-3.32876	-2.48964	0.93503
H	-2.69593	-0.94695	1.50803
C	-0.49336	-2.12750	0.83720
C	0.85798	-2.32347	0.15038
H	-0.75497	-3.05076	1.37844
H	-0.41155	-1.31867	1.57825
C	2.02115	-2.21001	1.12466
H	0.97728	-1.52353	-0.61310
O	0.95024	-3.58667	-0.48527
C	3.38077	-2.22417	0.43577
H	1.96606	-3.02763	1.86096
H	1.92036	-1.25697	1.66787
C	4.50724	-1.86084	1.38879
H	3.35215	-1.46600	-0.37359
O	3.70415	-3.48924	-0.12156
C	5.88987	-1.76670	0.70128
H	4.52975	-2.61382	2.19155
H	4.26199	-0.89395	1.85341
C	6.88602	-2.83173	1.16206
H	6.32958	-0.79001	0.93549
O	5.77631	-1.76888	-0.72659
C	8.30443	-2.55368	0.64365
H	6.56175	-3.83010	0.82927
H	6.91430	-2.83887	2.26135
C	9.37008	-3.48525	1.21426
O	8.57247	-1.22599	1.07461
O	8.37411	-2.65018	-0.75051
C	10.75162	-2.99600	0.80864
H	9.21042	-4.50773	0.84731
H	9.29560	-3.49555	2.31166
C	10.96623	-1.54647	1.26158
H	10.81756	-3.01187	-0.29416
O	11.71366	-3.85843	1.36691

C	9.82054	-0.69447	0.66347
C	12.36813	-1.03220	0.86061
H	10.88365	-1.48741	2.35973
C	9.79894	0.77010	1.09853
C	9.13283	1.75546	0.11455
H	10.81776	1.09247	1.34440
H	9.22725	0.81985	2.03691
C	7.72713	1.36135	-0.21808
C	6.63470	1.95868	0.28699
C	5.29009	1.50294	0.03054
C	4.17538	2.01625	0.60863
C	2.85910	1.51469	0.34575
C	1.70556	1.94451	0.92322
C	-1.11495	2.75652	1.98253
C	-2.35470	3.05623	2.45762
C	-3.57005	2.32309	2.23262
C	-4.79065	2.76208	2.62076
C	-6.02759	2.04494	2.42116
C	-7.23405	2.57330	2.68866
C	-8.60056	1.95135	2.56821
C	-8.57620	0.55854	1.93559
H	-9.19745	2.60435	1.90557
C	-9.26087	1.96284	3.95320
O	-8.03459	0.77290	0.60778
C	-9.90746	-0.20064	1.77537
H	-7.87104	-0.08573	2.48575
O	-7.10095	-1.27219	0.62355
H	-1.31582	-0.83005	-0.65553
H	-5.93731	1.02519	2.03140
H	-7.25443	3.59786	3.07966
H	-3.50819	1.35380	1.72748
H	-4.86483	3.74032	3.10864
C	-0.79828	1.69409	1.07365
H	-2.45353	3.94812	3.08285
H	2.78953	0.70792	-0.39430
H	5.18362	0.66058	-0.66382
H	4.27012	2.84367	1.31983
H	7.61623	0.48860	-0.87072
H	6.75266	2.82154	0.95202
H	9.87080	-0.76943	-0.43985
H	0.14864	-3.69055	-1.02561
H	2.90017	-3.83378	-0.54992
H	5.25383	-2.56070	-0.95918
H	7.56316	-2.23412	-1.09783
H	12.55542	-3.56807	0.97609
O	12.76862	0.04875	1.32353
O	12.99314	-1.73002	-0.01051
H	-8.81555	1.20184	4.60939
H	-10.34142	1.78978	3.89771
H	-9.11149	2.93899	4.43298
C	-11.00537	0.67914	1.17226
C	-10.34709	-0.93252	3.04561
H	-9.66595	-0.98821	1.04023
C	-12.24289	-0.10183	0.74395
H	-10.59603	1.20867	0.29543
H	-11.31847	1.45526	1.88705
C	-13.27927	0.78502	0.06788
C	-14.48729	-0.02467	-0.40482
H	-12.81809	1.26179	-0.82032
O	-13.66209	1.76483	1.00328
C	-15.52215	0.83520	-1.11207
H	-14.96319	-0.50851	0.46463
H	-14.16742	-0.83593	-1.07781
C	-16.68744	0.18633	-1.74823
C	-16.91272	-1.19590	-1.70402
C	-18.02031	-1.75969	-2.31011

**C**	-18.94802	-0.95198	-2.98894
**C**	-18.72728	0.43698	-3.03436
**C**	-17.62063	0.98815	-2.42314
**H**	-16.21356	-1.85192	-1.18383
**H**	-18.18397	-2.83708	-2.26012
**N**	-20.07836	-1.50030	-3.55056
**H**	-19.44444	1.07483	-3.55353
**H**	-17.44665	2.06402	-2.45586
**H**	-20.05485	-2.49242	-3.75849
**H**	-20.54866	-0.95347	-4.26327
**O**	-15.38454	2.05256	-1.15095
**H**	-14.24302	2.37674	0.52194
**H**	-10.85274	-0.27629	3.76446
**H**	-9.49100	-1.39881	3.55293
**H**	-11.04944	-1.73806	2.79621
**C**	11.17675	2.14177	-1.06048
**C**	11.93500	0.89934	-1.51240
**C**	13.40616	1.18640	-1.36399
**O**	11.69878	0.60903	-2.87223
**H**	11.64741	0.05039	-0.89422
**C**	13.76490	2.35095	-2.26320
**H**	13.62638	1.41782	-0.31224
**N**	14.16964	-0.05734	-1.66148
**C**	12.86194	3.54374	-1.85773
**H**	13.55825	2.09236	-3.31690
**O**	15.13621	2.61165	-2.05157
**O**	11.48767	3.20775	-1.92227
**C**	13.06868	4.74402	-2.75687
**H**	13.13556	3.80297	-0.81373
**H**	10.73719	0.53567	-2.98659
**H**	15.45466	3.22046	-2.73287
**H**	15.17525	0.13566	-1.68141
**H**	13.91922	-0.75428	-0.94388
**H**	12.82430	4.48740	-3.79624
**H**	14.10446	5.10361	-2.70513
**H**	12.41173	5.56340	-2.44431
**H**	11.47812	2.40946	-0.02842
**O**	9.82016	1.87039	-1.14665
**H**	9.13127	2.75396	0.59113
**H**	13.87411	-0.43221	-2.57617
**H**	-11.95805	-0.91568	0.05676
**H**	-12.72011	-0.57148	1.61911
**O**	-1.68188	-2.82258	-1.08565
**H**	-2.15501	-2.51745	-1.87152
**O**	-6.09643	-1.04383	-3.11440
**C**	0.43535	1.36781	0.59605
**H**	1.75266	2.74797	1.66365
**H**	-0.29419	3.40893	2.28884
**H**	-1.64019	1.11402	0.68537
**H**	0.48094	0.56284	-0.14467
**H**	-4.71575	-0.34750	0.54301
**H**	-3.53122	0.12469	-0.67512

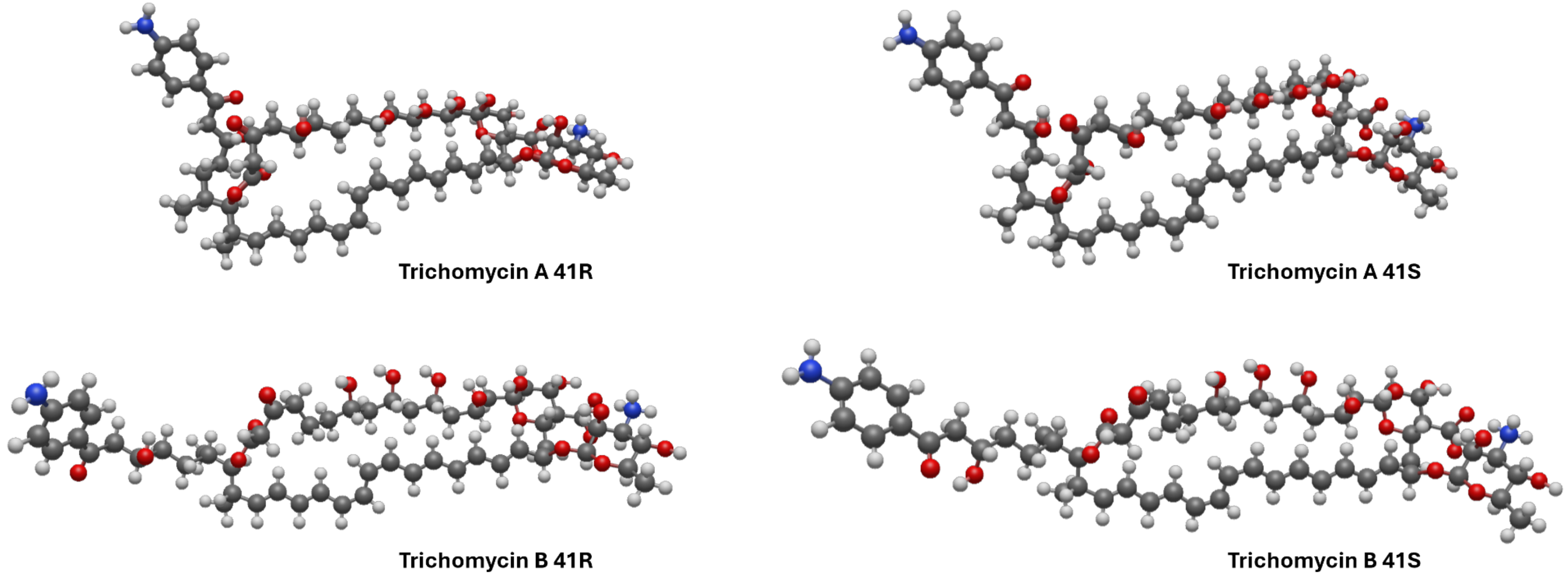

Figure S16 3D structures for both enantiomers of trichomycin A and B.

SUPPLEMENTARY INFORMATION FOR THE HLA EQUATION

Table *S4*. Parameters for HLA equation used for trichomycin A and B. ‘S1-S4’ stand for ‘substituents 1-4’.

Epimer	Torsion Angle	S1	S2	S3	S4	ξ_1	ξ_2	ξ_3	ξ_4	$\Delta\chi_1$	$\Delta\chi_2$	$\Delta\chi_3$	$\Delta\chi_4$	P1	P2	P3	P4	P5	P6
**R**	H40a–H41	$CH_2C(O)R$	OH	CH_2CH_2R	H	1	-1	1	-1	0,72	1,33	0,5	0	14,64	-0,78	0,58	0,34	-2,31	18,4
**R**	H40b–H41	$CH_2C(O)R$	OH	H	CH_2CH_2R	1	-1	1	-1	0,72	1,33	0	0,5						
**S**	H40a–H41	OH	$CH_2C(O)R$	CH_2CH_2R	H	1	-1	1	-1	1,33	0,72	0,5	0						
**S**	H40b–H41	OH	$CH_2C(O)R$	H	CH_2CH_2R	1	-1	1	-1	1,33	0,72	0	0,5						
**R**	H41–H42a	C(O)R	H	OH	CH_2CH_2R	1	-1	1	-1	0,72	0	1,33	0,5						
**R**	H41–H42b	H	C(O)R	OH	CH_2CH_2R	1	-1	1	-1	0	0,72	1,33	0,5						
**S**	H41–H42a	C(O)R	H	CH_2CH_2R	OH	1	-1	1	-1	0,72	0	0,5	1,33						
**S**	H41–H42b	H	C(O)R	CH_2CH_2R	OH	1	-1	1	-1	0	0,72	0,5	1,33						

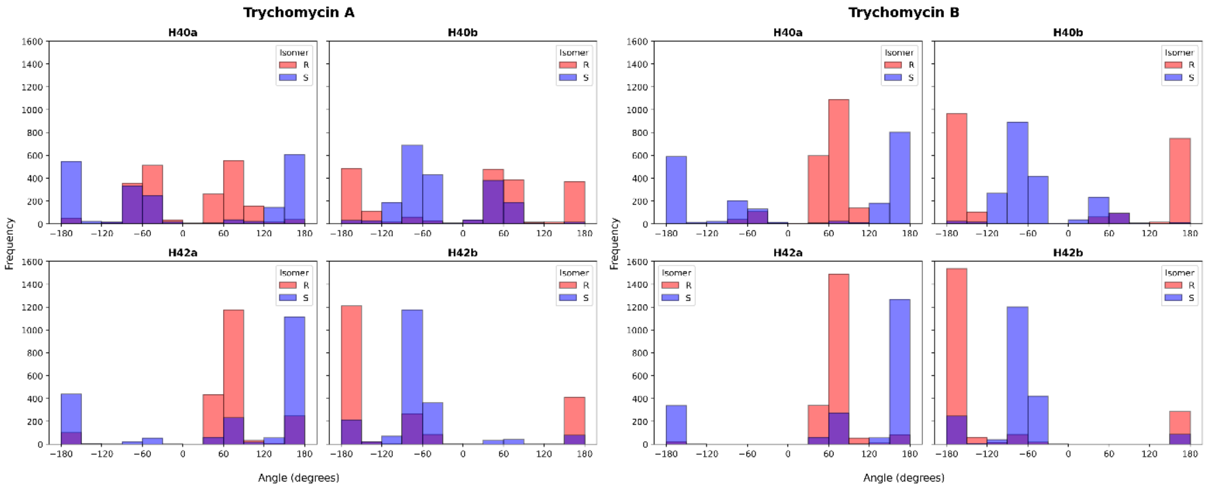

Figure *S17*. Distribution of the four dihedral angles during the 500 ns equilibrium MD simulations of the both possible trichomycin A enantiomers (left), distribution of the four dihedral angles monitored of the both possible trichomycin B enantiomers (right).

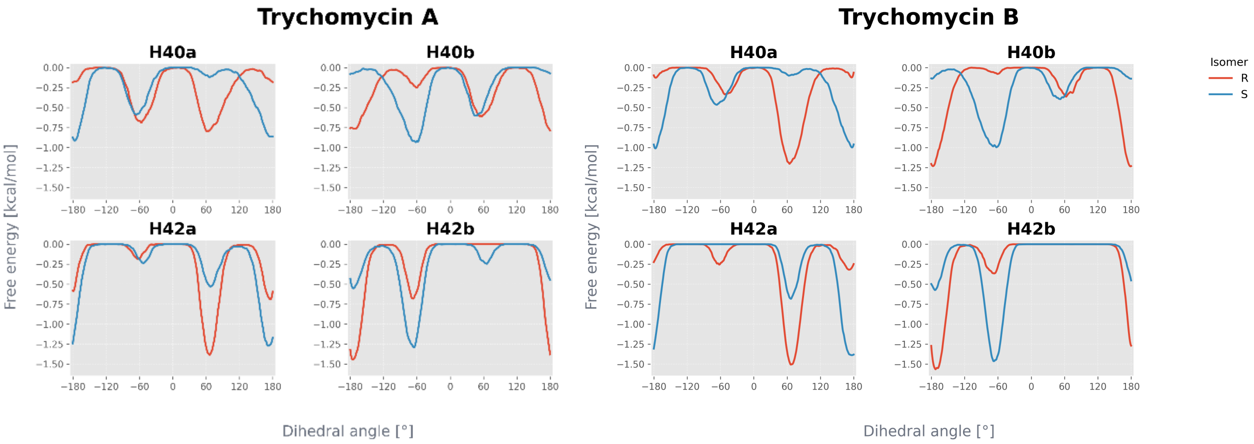

Figure *S18*. Free energy profiles for all monitored dihedral angles for both enantiomers of trichomycin A (left) *and* trichomycin B (right).

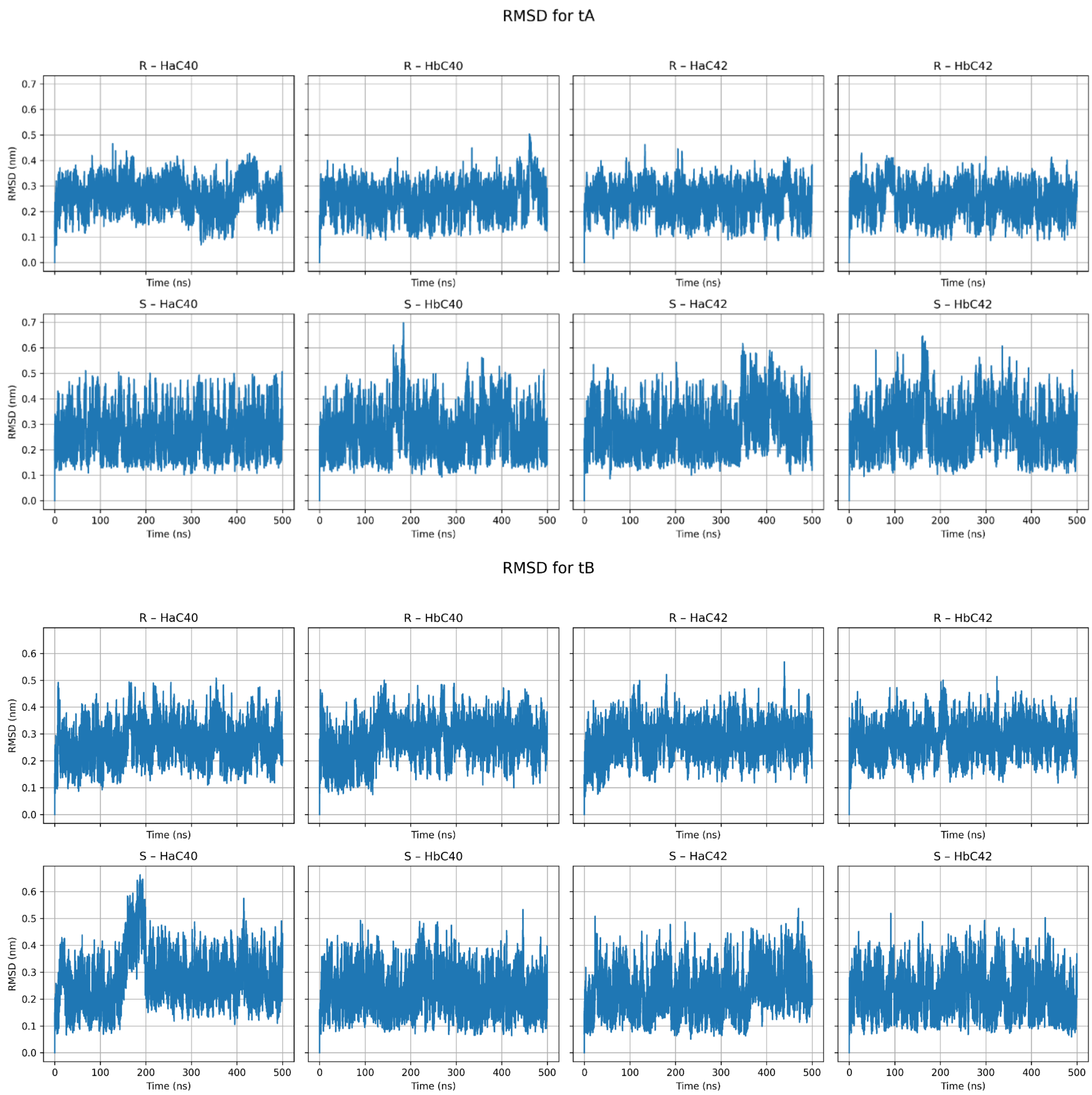

Figure S19 Time-dependent RMSD plots for both enantiomers of trichomycin A (upper) and trichomycin B (lower).

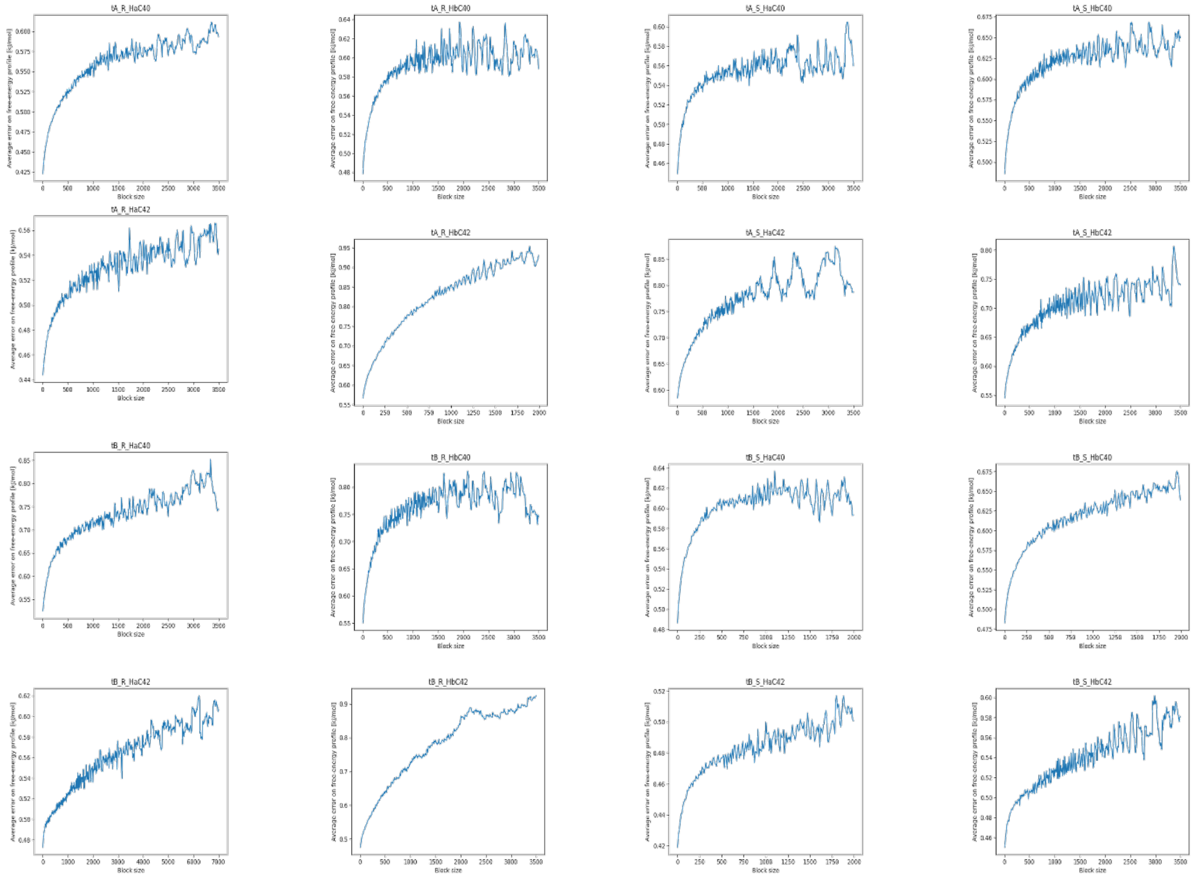

Figure S20 Block-averaged free energy errors for both enantiomers of trichomycin A (row 1 and 2) and trichomycin B (row 3 and 4).

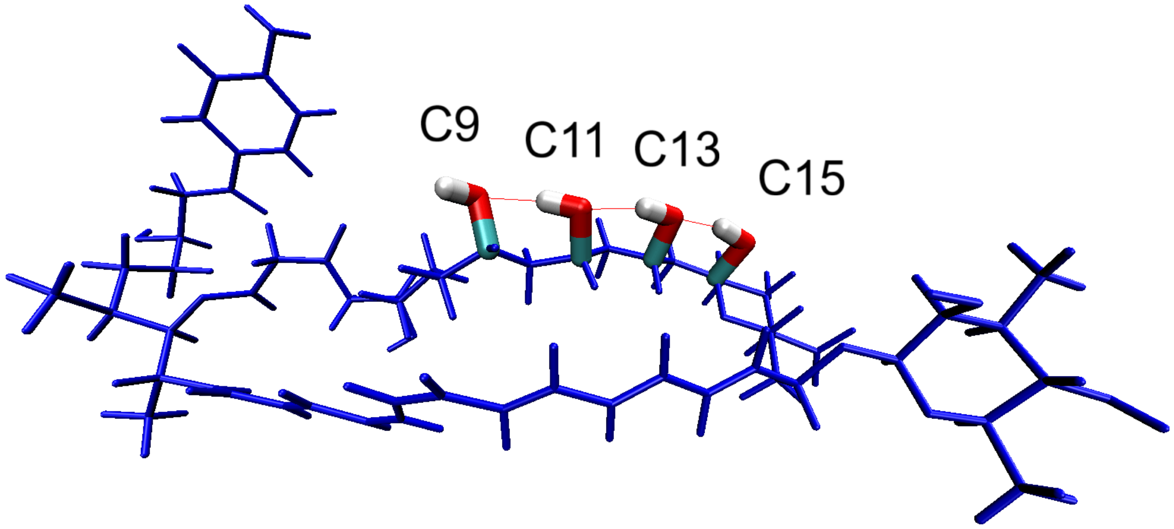

Figure S21 C9-C15 hydrogen bonding network of Trichomycin A.

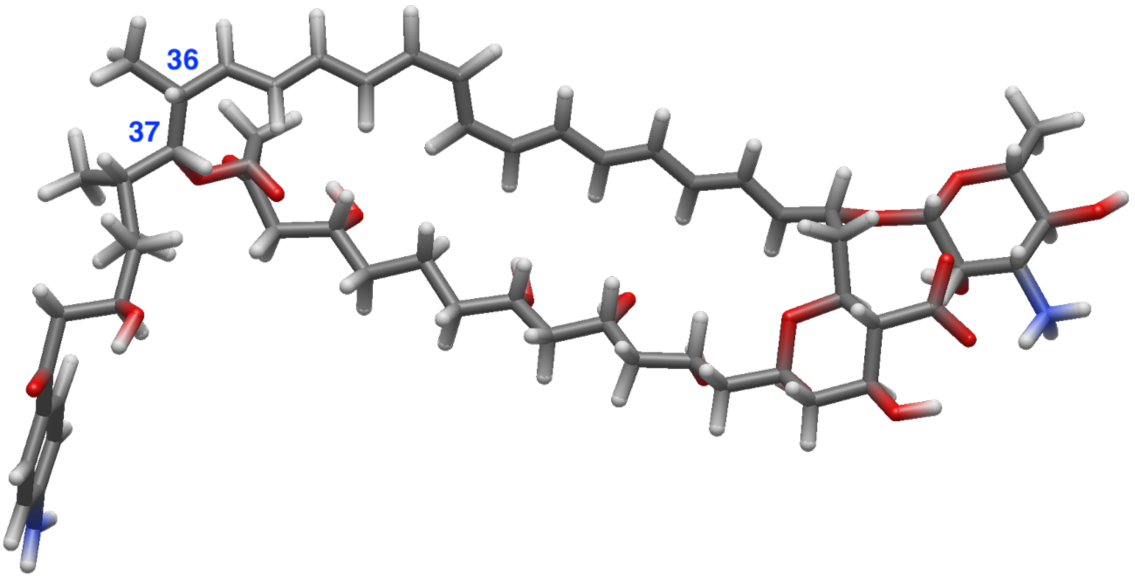

Figure S22 Trichomycin A with absolute configuration inversed at C36 and C37.
